# Calreticulin-targeting L-asparaginase-flagellin conjugate enhances *Salmonella*-mediated antitumor efficacy

**DOI:** 10.1038/s41420-026-03300-x

**Published:** 2026-08-09

**Authors:** Dinh-Huy Nguyen, Aqeel Rana Afzal, Phuong Thi-Minh Nguyen, Quoc-Thai Do-Ba, Khuynh Van Nguyen, Wan-Sik Lee, Jung-Joon Min, Yeongjin Hong

**Affiliations:** 1 Institute for Molecular Imaging and Theranostics, Chonnam National University, Jeonnam, Republic of Korea; 2 Department of Nuclear Medicine, Chonnam National University Medical School and Hwasun Hospital, Jeonnam, Republic of Korea; 3 Institute of Biomedical Science, Chonnam National University Hwasun Hospital, Jeonnam, Republic of Korea; 4 Department of Biomedical Science (BrainKorea21 Plus) Chonnam National University Graduate School, Jeonnam, Republic of Korea; 5 Division of Gastroenterology, Department of Internal Medicine, Chonnam National University Medical School and Hwasun Hospital, Jeonnam, Republic of Korea; 6 CNCure Co. Ltd., Jeonnam, Republic of Korea; 7 Department of Microbiology and Immunology, Chonnam National University Medical School, Jeonnam, Republic of Korea

**Keywords:** Cancer microenvironment, Drug delivery, Drug development, Cancer metabolism

## Abstract

Targeted therapeutics have transformed cancer treatment by selectively eliminating malignant cells while limiting systemic toxicity. L-asparaginase (L-ASNase), which induces metabolic stress by depleting asparagine (Asn), is clinically used for hematological malignancies but shows limited activity against solid tumors due to poor delivery and an immunosuppressive microenvironment. We previously developed CRT3LP, a calreticulin (CRT)-targeting monobody-L-ASNase fusion protein, designed to exploit immunogenic cell death (ICD); however, its therapeutic potential is constrained by insufficient immune activation. Here, we show that CRT3LFP, a multifunctional fusion protein incorporating the flagellin B subunit (FlaB) into the CRT3LP scaffold, effectively promotes M2-to-M1 macrophage polarization while maintaining tumor-selective metabolic disruption. In combination with the tumor-colonizing bacterial strain CNC018, which induces surface-exposed CRT, CRT3LFP achieves precise tumor localization. This synergistic approach significantly inhibits tumor growth and reshapes the tumor microenvironment, characterized by enhanced macrophage polarization, dendritic cell maturation, and CD8^+^ T-cell expansion. Additionally, CD47 blockade further potentiates this effect, fostering the formation of durable immune memory. Together, our findings establish CRT3LFP as a tumor-targeted immunometabolic platform that integrates metabolic deprivation with coordinated innate and adaptive immune activation to overcome resistance in solid tumors.

**Figure:**
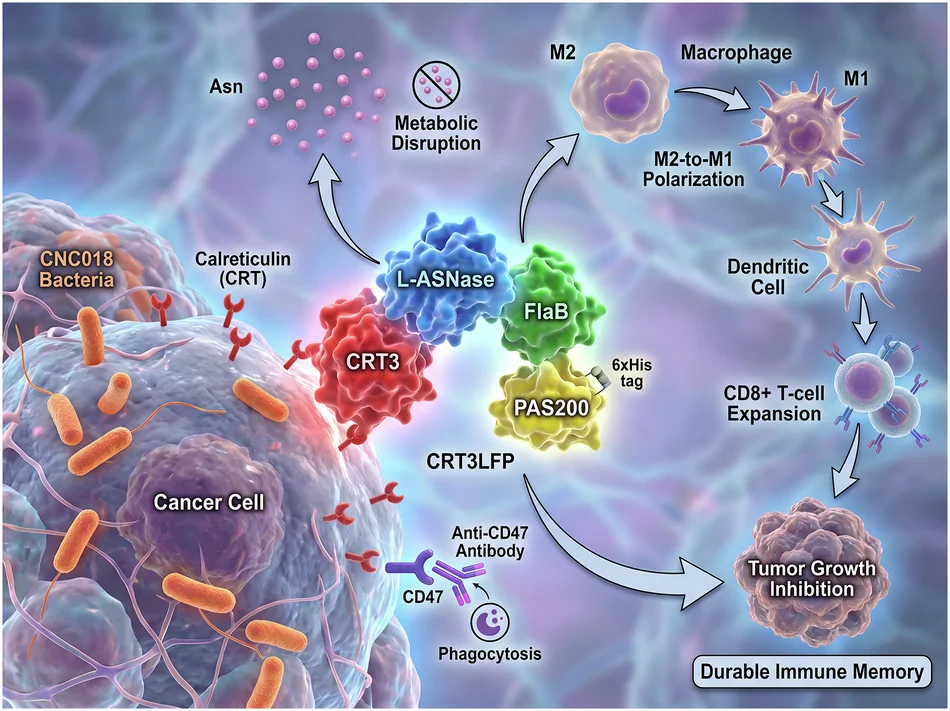

## Introduction

Targeted therapies that selectively eliminate tumor cells while limiting systemic toxicity have transformed cancer treatment [1]. However, responses remain limited in many settings owing to poor intratumoral drug exposure, suboptimal targeting, and insufficient activation of antitumor immunity [2–5]. A promising approach to improve the efficacy of targeted agents is to reprogram the tumor microenvironment (TME) to promote robust innate and adaptive immunity.

Radiotherapy and chemotherapy remain cornerstones of cancer treatments that often induce immunogenic cell death (ICD), characterized by the translocation of calreticulin (CRT) to the surface of dying tumor cells. Surface-exposed CRT acts as an “eat-me” signal that promotes recognition and phagocytosis of tumor cells by antigen-presenting cells [6, 7]. Despite these immunogenic features, conventional cytotoxic therapies can also induce an immunosuppressive TME, for instance, by promoting the polarization of M2-like tumor-associated macrophages (TAMs), which facilitate tumor progression and inhibit antitumor immunity [8, 9]. Moreover, therapeutic efficacy can be reduced in hypoxic tumor regions, and off-tumor toxicity that limits the dose remains a major concern [10–12].

Bacteria-mediated cancer therapy has emerged as an alternative strategy to overcome these limitations by using attenuated strains of *Salmonella typhimurium* (*S. typhimurium*), *Escherichia coli* (*E. coli*), *Clostridium*, *Listeria*, and *Bifidobacterium*, among others [13–21]. These bacteria are uniquely capable of targeting, colonizing, and actively proliferating within hypoxic and necrotic tumor niches that are typically inaccessible to conventional therapeutic agents [13, 21, 22]. Among various strains, the attenuated *S. typhimurium* CNC018 has emerged as a promising platform to induce tumor cell death and reprogram the TME for enhanced tumor control [23–25]. However, the potential of bacteria to function as a biological “primer”, specifically through the induction of surface CRT exposure to facilitate high-affinity engagement of subsequent targeted therapies, remains unexplored.

Among metabolic interventions, *E. coli* L-asparaginase (L-ASNase), an enzyme that hydrolyzes the amino acid asparagine (Asn), is an essential therapeutic agent for hematologic malignancies, particularly acute lymphoblastic leukemia (ALL), in which leukemic blasts depend on extracellular Asn for survival [26]. Moreover, Asn deprivation has been reported to enhance the metabolic fitness and antitumor activity of CD8^+^ T cells [27]. Nevertheless, the therapeutic application of L-ASNase in solid tumors has been limited by inadequate tumor selectivity and insufficient immunostimulatory activity [28, 29]. To address these challenges, we previously developed CRT3LP, a PASylated L-ASNase conjugate fused to CRT3, a CRT-binding monobody that targets CRT exposed on tumor cells following ICD induced by chemotherapy or radiotherapy [6, 30, 31]. Although CRT3-mediated targeting and PASylation improved delivery, pharmacokinetics, and the efficacy of chemo- or radiotherapy, the overall benefit remained constrained by limited immune activation within the TME [30, 31].

While immune checkpoint therapies have revolutionized oncology, their efficacy is often limited by an immunosuppressive TME. Tumor-associated macrophages (TAMs) predominantly exhibit a pro-tumoral M2-like phenotype; with only a small population of an antitumoral M1-like phenotype, they can promote tumor growth, angiogenesis, and metastasis [32, 33]. Consequently, reprogramming TAMs toward an M1-like phenotype has emerged as a promising immunotherapeutic strategy [17, 34, 35]. Flagellin B (FlaB), a flagellar subunit from *Vibrio vulnificus*, is a potent Toll-like receptor 5 (TLR5) agonist reported to elicit robust antitumor activity in primary and metastatic settings by promoting M2-to-M1 macrophage polarization [17, 18, 36]. In parallel, tumor cells often evade innate clearance by overexpressing CD47, a “don’t-eat-me” signal that inhibits macrophage-mediated phagocytosis via SIRPα receptor [37]. Targeted blockade of the CD47-SIRPα axis using an anti-CD47 antibody (αCD47 Ab) can restore phagocytic capacity and support the cross-priming of CD8^+^ T cells, potentially synergizing with myeloid-reprogramming agents to drive robust antitumor immunity.

**Fig. 1:**
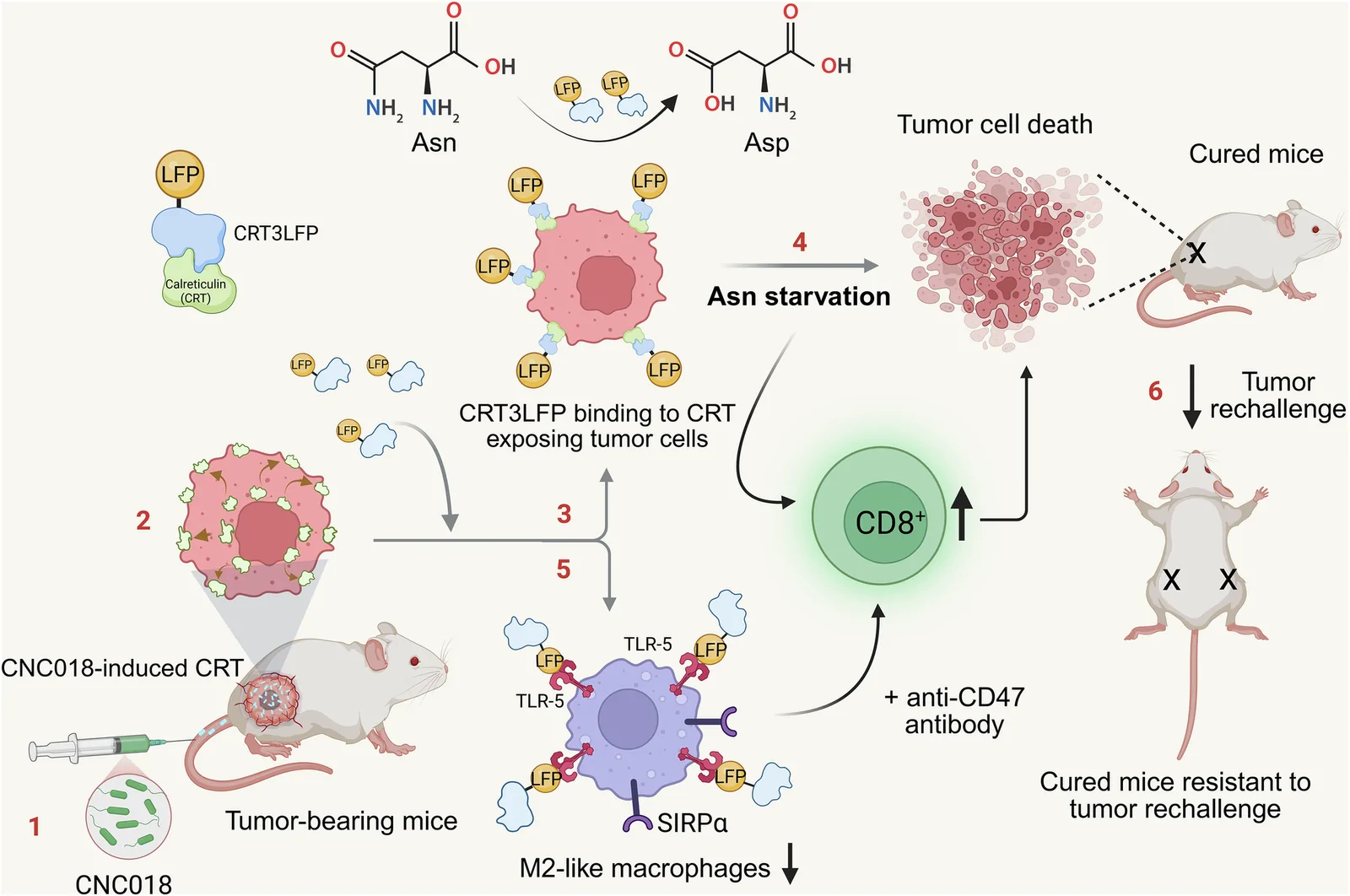
Schematic illustration of the enhanced tumor-specific targeting and antitumor effects of the PASylated calreticulin (CRT)-targeted L-asparaginase (L-ASNase)-flagellin B (FlaB) fusion protein (CRT3LFP) in tumor-bearing mice treated with *Salmonella* CNC018. 1. Following *i.v*. injection (via the tail vein) into tumor-bearing mice, CNC018 bacteria selectively localize to tumor tissue and induce the exposure of CRT on the surface of tumor cell membranes. 2. Upon *i.p*. injection, CRT3LFP specifically targets CRT-exposing tumor cells. 3. The L-ASNase activity of CRT3LFP depletes asparagine (Asn) within the tumor microenvironment (TME) by converting Asn to aspartate (Asp). 4. Asn depletion inhibits tumor cell proliferation and enhances CD8^+^ T cell-mediated antitumor responses. 5. Simultaneously, the FlaB moiety of CRT3LFP binds to Toll-like receptor 5 (TLR5) on macrophages, promoting their polarization from an M2-like to an M1-like phenotype. 6. Mice cured by CNC018 and CRT3LFP combination establish antitumor immune memory and exhibit resistance to tumor rechallenge in combination with αCD47 Ab. Figure created with BioRender.com.

Here, we engineered CRT3LFP, a multifunctional fusion protein that integrates FlaB into the CRT3LP scaffold to couple tumor-selective metabolic disruption with potent innate immune activation. Our findings demonstrate that the attenuated *S. typhimurium* strain CNC018 acts as a biological primer, inducing widespread surface CRT exposure to facilitate precise tumor localization of CRT3LFP. Notably, this combination effectively remodels the TME, drives M2-to-M1 macrophage polarization, and expands CD8^+^ T cells. Finally, we establish that a triple combination, integrating CNC018, CRT3LFP, and CD47 blockade, effectively disrupts the “don’t eat me” signal to achieve synergistic tumor regression and the induction of durable immune memory (Fig. 1).

## Results

### Engineering and characterization of CRT3LFP

**Fig. 2:**
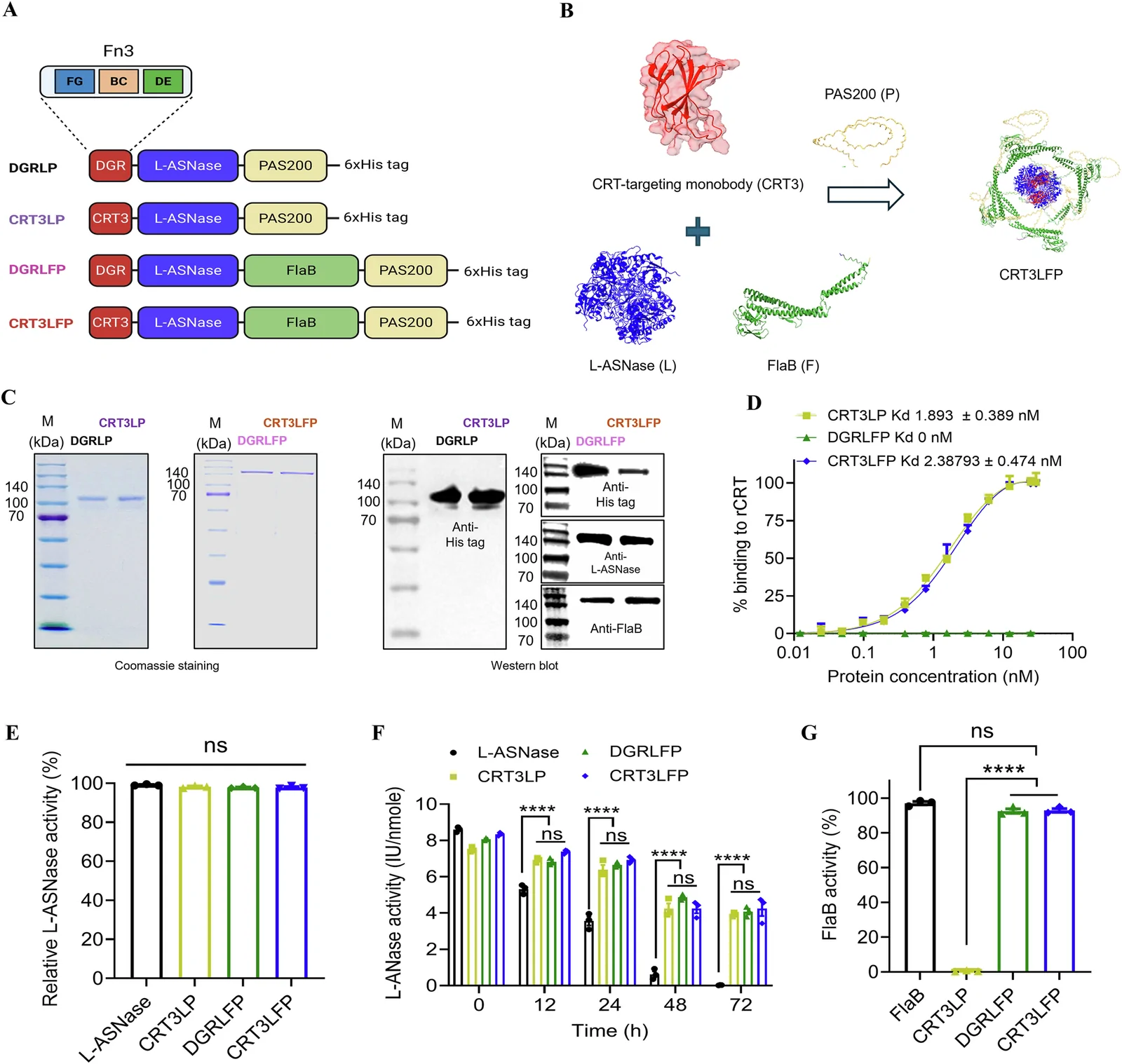
Engineering and characterization of the PASylated CRT3–L-ASNase–FlaB fusion protein (CRT3LFP). **A** Schematic of the open reading frames (ORFs) for the DGRLP, CRT3LP, DGRLFP, and CRT3LFP constructs. Each construct encodes an N-terminal monobody, L-asparaginase (L-ASNase, L) fused to FlaB (F), and a C-terminal 6×His-tagged PAS moiety (P). The CRT-targeting monobody (CRT3) contains the sequences KLGFFKR and GQPMYGQPMY in the BC and FG loops, respectively, within a human fibronectin type III (Fn3) domain backbone. The DGR monobody is negative control, carrying a DGR sequence in place of RGD in the FG loop. Figure created with BioRender.com. **B** Computational model of CRT3LFP. Because L-ASNase (blue) forms a tetramer, four CRT3 monobody units (red) and four FlaB domains (green) are appended to the tetramer’s N- and C-termini, respectively. Four 6×His-tagged PAS moieties (yellow) with flexible conformations are attached to the C-termini of the FlaB domains. **C** Purification of DGRLP, CRT3LP, DGRLFP, and CRT3LFP. pET-based plasmids carrying each ORF were transformed into *E. coli* BL21(DE3), induced with IPTG, and proteins purified by Ni-NTA affinity chromatography. Samples were analyzed by SDS-PAGE and Coomassie staining (left). Proteins transferred to membranes were probed by Western blot with anti-His, anti-L-ASNase, and anti-FlaB antibodies (right). M, molecular weight marker (kDa). **D** Binding affinity of CRT3LFP for calreticulin (CRT). Increasing concentrations of CRT were incubated on immobilized CRT3LFP and binding measured by ELISA (OD450). Affinity is reported as the dissociation constant (*K*_D_). **E** L-ASNase activity of CRT3LFP. Specific enzyme activity (IU per nmol of protein) was measured and compared with native L-ASNase (*n* = 3 technical replicates; ns, not significant; unpaired two-tailed *t*-test). **F** Serum stability of CRT3LFP. 60 μg CRT3LP or 90 μg CRT3LFP (each corresponding to 8 IU per nmol) were mixed with an equal volume of mouse serum and incubated at 37 °C. Residual L-ASNase activity was measured at the indicated time points. Native L-ASNase was included as a control. **G** Functional FlaB activity of CRT3LFP. HEK293 cells stably expressing mouse TLR5 and an NF-κB-inducible SEAP reporter (HEK293/mTLR5, pNiFty2-SEAP) were treated with purified proteins (4 nM) for 6 h. FlaB function was assessed by SEAP alkaline phosphatase activity; purified FlaB served as a positive control (*n* = 3 technical replicates; ****P < 0.0001; ns, not significant; unpaired two-tailed *t*-test).

The genes of PASylated CRT3-targeting L-ASNase (CRT3LP) and its negative-control variant (DGRLP) were genetically fused with FlaB (Fig. 2A), named CRT3LFP and DGRLFP, respectively. In them, an open reading frame (ORF) of the FlaB moiety was sequentially integrated between the L-ASNase and PAS200 tag in the CRT3LP and DGRLP genes. In computational modeling analysis based on the CRT3LP structure previously depicted [30, 31], the FlaB moiety did not interfere with the overall stability of the CRT3 monobody and L-ASNase structure in CRT3LFP (Fig. 2B). Consequently, four moieties of the CRT3 monobody and the FlaB were predicted to be conjugated to a tetrameric L-ASNase. Furthermore, PAS200 tags located at the C-termini of the FlaB moieties were predicted to be exposed to the outside of the enzyme complex, thereby protecting the enzyme complex from proteolytic degradation. Notably, a His-tag located on the C-terminal end of PAS200 tags facilitated efficient protein purification via immobilized metal affinity chromatography.

We purified CRT3LFP and DGRLFP following IPTG-induced expression in *E. coli* transformed with their expression vectors and characterized the products via SDS-PAGE and immunoblotting (Fig. 2C). The calculated molecular weights of a single subunit were approximately 64 kDa for DGRLP or CRT3LP and 105 kDa for DGRLFP or CRT3LFP, respectively. However, as observed by SDS-PAGE, the protein migrated at higher apparent molecular weights, exceeding 100 kDa for DGRLP or CRT3LP and 140 kDa for DGRLFP or CRT3LFP, respectively (Fig. 2C, left). This discrepancy is consistent with previous reports indicating that PASylation increases the hydrodynamic volume of proteins, thereby reducing their electrophoretic mobility [31]. The purification and identification of DGRLFP and CRT3LFP were further confirmed by immunoblotting using antibodies targeting the His-tag, L-ASNase, and FlaB moieties (Fig. 2C, right).

We assessed the binding affinity of CRT3LFP against CRT by ELISA assay. The dissociation constant (K_D_) values of CRT3LFP and CRT3LP were 2.388 nM and 1.893 nM, respectively, indicating that FlaB integration had a negligible effect on the CRT affinity of CRT3LFP, consistent with our computational modeling predictions. The control protein DGRLFP exhibited no detectable binding to CRT (Fig. 2D). Surface plasmon resonance (SPR) analysis further confirmed that CRT3LFP possessed nanomolar affinity for CRT with a K_D_ value of 4.52 nM (Fig. S1). Next, we evaluated the L-ASNase activity of the engineered proteins (Fig. 2E). The activity of CRT3LFP was comparable to that of native L-ASNase, CRT3LP, and DGRLFP, demonstrating that the inclusion of FlaB moieties did not compromise the enzymatic function of the L-ASNase core. To assess the impact of PASylation on stability, we incubated CRT3LFP in mouse serum at 37 °C (Fig. 2F). After 48 h, approximately 50% of the initial L-ASNase activity was retained in CRT3LFP, as well as in the other PASylated variants (CRT3LP and DGRLFP), whereas only 7.25% of activity remained for non-PASylated L-ASNase. This indicates that the PAS tag effectively protects the protein complex from systemic proteolytic degradation. Finally, the functional activity of the FlaB moiety was measured in HEK293/mTLR5 cells transfected with pNiFty(2)-SEAP (Fig. 2G). Robust luminescence signals resulting from TLR5 activation were similarly observed following treatment with CRT3LFP, the FlaB-containing control DGRLFP, and purified FlaB protein, whereas no such signal was detected in cells treated with CRT3LP. These results confirm that the FlaB moiety within the CRT3LFP construct retains its capacity to activate TLR5 signaling at a level comparable to that of free FlaB.

### Binding of CRT3LFP to CRT and its cytotoxicity in CNC018-pretreated tumor cells

**Fig. 3:**
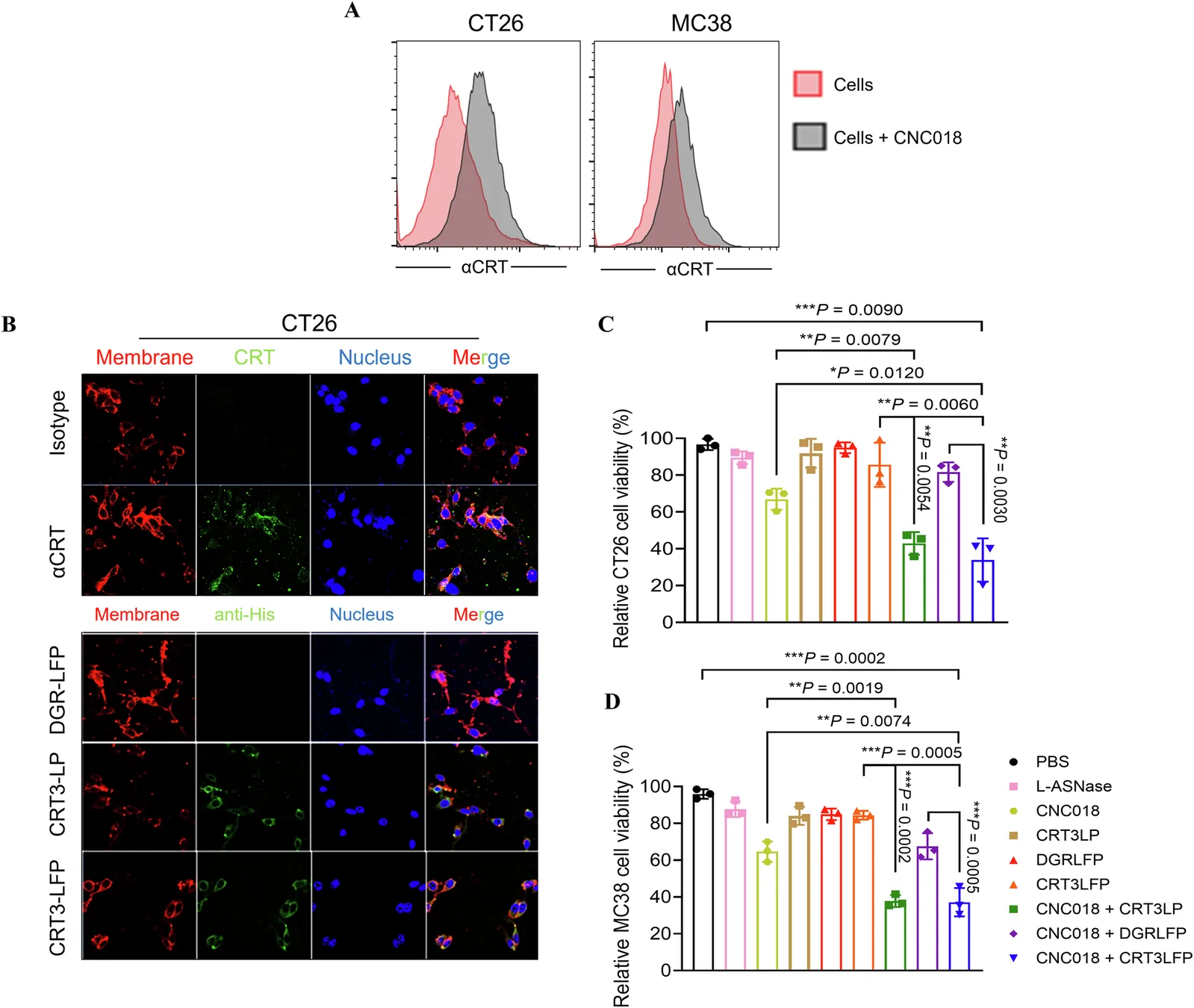
Binding of CRT3LFP to calreticulin (CRT) exposed on tumor cells following CNC018 treatment. **A** Flow cytometry of tumor cells treated with CNC018. CT26 (left) and MC38 (right) cells were incubated with CNC018 (MOI 10) for 20 h, harvested, and stained with anti‑CRT antibody prior to flow cytometric analysis. An isotype control antibody was included. **B** Immunofluorescence of CT26 cells treated with CNC018. Cells were treated with CNC018 (MOI 10) for 20 h, fixed (no permeabilization), and stained with anti‑CRT primary antibody followed by Alexa Fluor 488‑conjugated secondary antibody (green). As a separate detection, cells were incubated with purified proteins (CRT3LP, DGRLFP, or CRT3LFP), then stained with anti‑His monoclonal antibody (1:1,000) and Alexa Fluor 488‑conjugated secondary antibody (5 μg/mL) for 1 h. Cell membranes and nuclei were co‑stained with WGA (red) and DAPI (blue), respectively. Images were captured by confocal microscopy (40_×_). An isotype control served as the antibody control. **C** Viability of CT26 cells co‑treated with CNC018 and CRT3LFP. CT26 cells (1 × 10^4^ cells/well) were exposed to CNC018 (MOI 10) in 1 mL culture medium for 20 h. After washing, cells were treated with purified protein (4 IU L‑ASNase) for 4 h, washed, incubated for 1 h, and then cell viability was measured. PBS was used as the vehicle control (*n* = 3 technical replicates; unpaired two‑tailed *t*‑test). **D** Viability of MC38 cells co-treated with CNC018 and CRT3LFP. Procedures were the same as in (**C**) for MC38 cells (*n* = 3 technical replicates; unpaired two‑tailed *t*‑test).

Given that *S. typhimurium* induces ICD in tumor cells [16], we hypothesized that CRT, a hallmark biomarker of ICD, would be exposed on the surface of treated tumor cells. Flow cytometric analysis confirmed that CRT expression was significantly elevated on the surface of mouse colon tumor cells (CT26 and MC38) following a 20-h in vitro treatment with CNC018 (Fig. 3A). CRT exposure on CT26 tumor cells was further confirmed by immunofluorescence staining (Fig. 3B). Consistent with our binding assays, CRT3LFP and CRT3LP exhibited specific binding to CNC018-treated CT26, whereas the non-targeting control protein DGRLFP showed no significant binding. These results demonstrate that the CRT3 monobody integrated into the CRT3LFP and CRT3LP constructs successfully and selectively targets CRT-exposed tumor cells induced by CNC018-mediated ICD.

We next evaluated the additive cytotoxic effects of CRT3LFP and CRT3LP on tumor cells pretreated with CNC018. Treatment with CRT3LP, CRT3LFP, DGRLFP, or L-ASNase alone had a minimal impact on the viability of CT26 cells; conversely, CNC018 treatment alone significantly reduced viability by approximately 35% (Fig. 3C). Notably, when cells were pretreated with CNC018, the subsequent addition of CRT3LFP or CRT3LP resulted in an approximately 2-fold further reduction in viability, respectively; this effect was not observed with DGRLFP control. Comparable antitumor effects were observed in MC38 cells pretreated with CNC018 (Fig. 3D). Collectively, these findings indicate that both CRT3LFP and CRT3LP selectively enhance the cytotoxicity of CNC018 in a CRT-dependent manner in tumor cells.

### Antitumor activity and safety evaluation of CRT3LFP in CT26 tumor-bearing mice

**Fig. 4:**
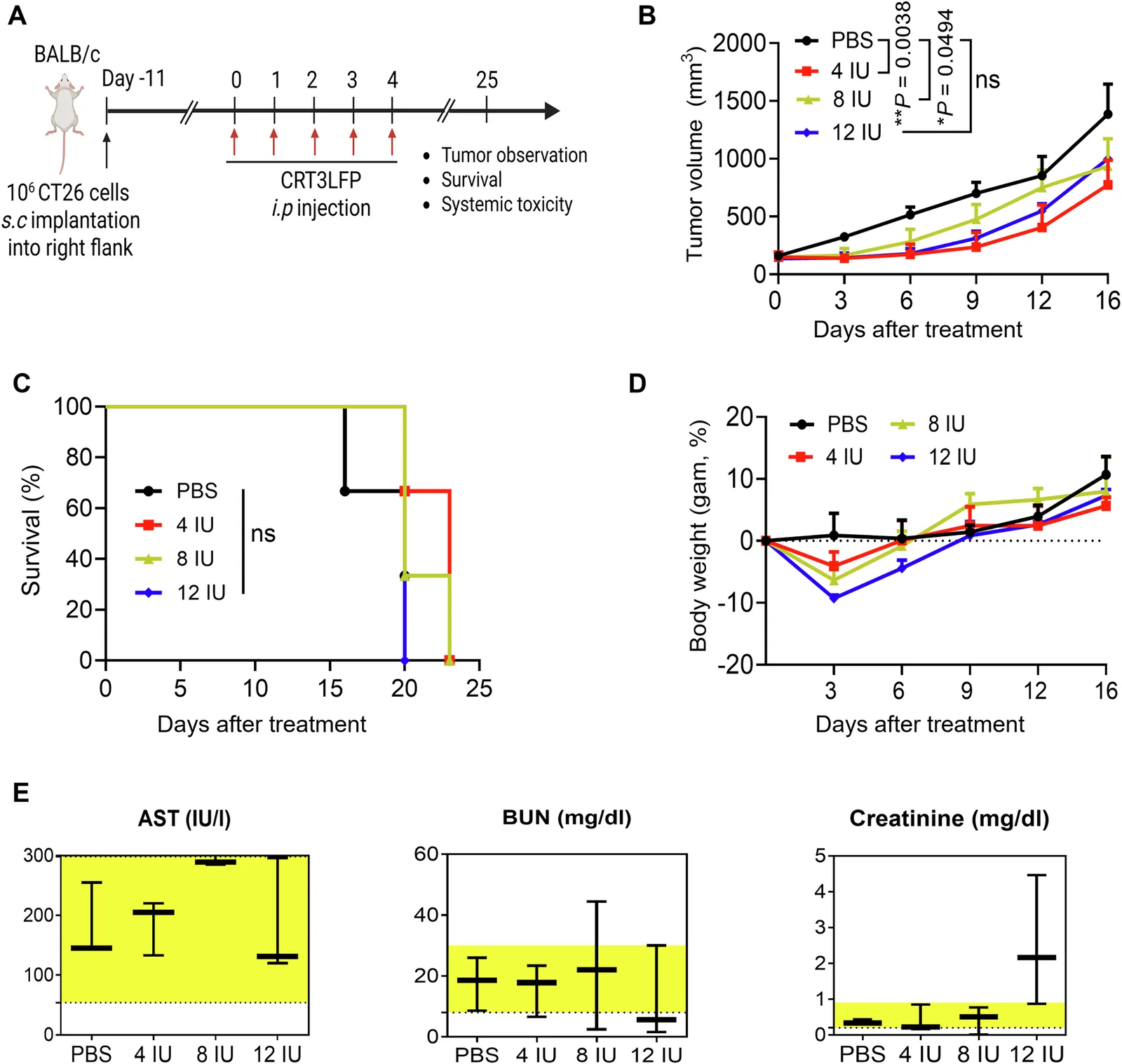
Antitumor efficacy and systemic safety of CRT3LFP in CT26 tumor-bearing mice. **A** Experimental scheme. BALB/c mice were inoculated subcutaneously (*s.c*.) with 1 × 10^6^ CT26 cells (black arrow). When tumors reached ~100–120 mm^3^ (day 0), mice were randomized into five groups. Indicated doses of CRT3LFP were administered intraperitoneally (*i.p*.) five times at 1-day intervals (red arrows). PBS was used as the vehicle control. Figure created with BioRender.com. **B** Mean tumor growth curves from (**A**) (*n* = 3 mice per group; ns, not significant; two-way ANOVA with Tukey’s multiple comparisons test). **C** Kaplan-Meier survival curves for each group (*n* = 3 mice/group; ns, not significant; log-rank [Mantel-Cox] test). **D** Body-weight changes during treatment (*n* = 3 mice/group; ns, not significant; two-way ANOVA with Tukey’s multiple comparisons test). **E** Systemic toxicity profile of CRT3LFP. On day 6, serum/plasma levels of aspartate aminotransferase (AST), blood urea nitrogen (BUN), and creatinine were measured. Box plots show the interquartile range (IQR); whiskers indicate the 10th and 90th percentiles; the central line denotes the median. Reference ranges are AST 54-298 IU/L, BUN 8-33 mg/dL, creatinine 0.2–0.9 mg/dL. Yellow shading denotes the normal range for each parameter, *n* = 3 mice/group.

To determine the optimal in vivo dose of CRT3LFP, we treated CT26 *s.c*. tumor-bearing BALB/c mice with five doses of CRT3LFP (4, 8, or 12 IU per injection) at 1-day intervals (Fig. 4A). While all doses elicited observable tumor growth inhibition and improved survival compared with the PBS-treated control group, the 4 IU dose provided the maximal therapeutic benefit (Fig. 4B, C, and Fig. S2A). Furthermore, the 4 IU regimen was better tolerated; these mice maintained the most stable body weight and exhibited no signs of systemic toxicity or treatment-related mortality (Fig. 4D, E, and Fig. S2B). In contrast, higher doses (8 and 12 IU) were associated with progressive body weight loss and diminished antitumor effectiveness. The dose-dependent decline in efficacy suggests that higher concentrations of CRT3LFP may elicit potent systemic TLR5 activation via the FlaB component before the CRT3 moiety can effectively localize and accumulate on CRT-exposed tumors. We hypothesize that such premature systemic immune activation induces a transient inflammatory response that narrows the therapeutic window and impairs the capacity of the protein to selectively reprogram the TME. Collectively, these results identify 4 IU per injection as the optimal dose to balance therapeutic potency with systemic safety.

### Antitumor activity of CRT3LFP in combination with CNC018

**Fig. 5:**
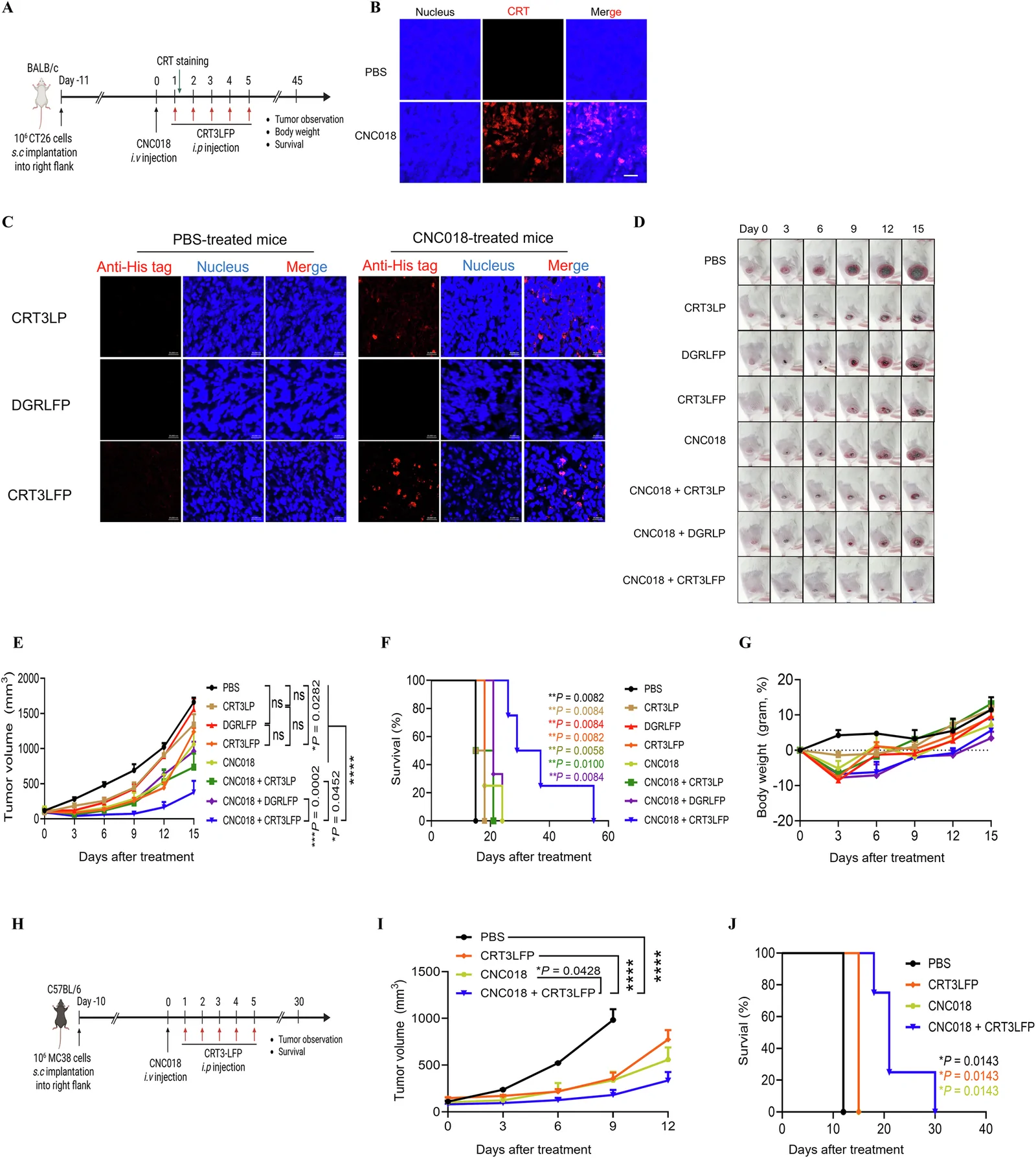
Therapeutic effects of CRT3LFP in tumor-bearing mice pretreated with CNC018 bacteria. **A** Experimental scheme for CT26 tumor-bearing mice treated with CNC018 + CRT3LFP (B-G). BALB/c mice were inoculated *s.c*. with CT26 cells (1 × 10^6^). When tumor volumes reached 100-120 mm³, mice were randomly divided into the indicated groups. CNC018 bacteria were *i.v*. administered on day 0 (black arrow). The proteins CRT3LP, DGRLFP, and CRT3LFP (4 IU/injection) were *i.p*. The treatment was administered five times starting from day 1, with 1-day intervals between each dose (red arrow). The tumor tissues were excised with 28 h post-bacterial injection to measure CRT exposure and protein targeting. Figure created with BioRender.com. **B** Representative immunohistochemical images show CRT exposure in excised CT26 tumor tissues. The nuclei and exposed CRT were stained with DAPI (blue) and anti-CRT antibody (red), respectively. The images were taken at 40× magnification. **C** Representative immunohistochemical images of CRT3LFP targeting CT26 tumor tissues treated with CNC018. The proteins and nuclei were co-stained with an anti-His-tag antibody (red) and DAPI (blue). The images were taken at 40× magnification. **D** Representative tumor images from (**A**). **E** Average growth curves of CT26 tumors from (A) (PBS, CRT3LP, DGRLFP, CRT3LFP, CNC018, CNC018 + CRT3LP, CNC018 + CRT3LFP, *n* = 4 mice/group; CNC018 + DGRLFP, *n* = 3 mice/group; ****P < 0.0001; ns, not significant; two-way ANOVA with Tukey’s multiple comparisons test). **F** Kaplan-Meier survival curves for CT26 tumor-bearing mice from (A) [Log-rank (Mantel-Cox) test]. **G** Changes in body weight (%) of CT26 tumor-bearing mice from A (two-way ANOVA with Tukey’s multiple comparisons test). **H** Experimental scheme for MC38 tumor-bearing mice treated with CNC018 + CRT3LFP (**I**–**J**). All procedures were followed as described in (**A**). Figure created with BioRender.com. **I** Average growth curves for MC38 tumors from (**H**) (PBS, *n* = 3 mice/group; CRT3LFP, CNC018, CNC018 + CRT3LFP, *n* = 4 mice/group; ****P < 0.0001; ns, not significant; two-way ANOVA with Tukey’s multiple comparisons test). **J** Kaplan-Meier survival curves for MC38 tumor-bearing mice from (**H**) [*n* = 3 mice/group; Log-ra*n*k (Mantel-Cox) test].

Bacteria possess the capacity to selectively colonize and proliferate within tumors, subsequently inducing ICD within the TME [16, 18]. Given that our previous work demonstrated that CRT3-based monobodies effectively target tumor tissues following ICD-induced surface translocation of CRT [15, 31], we investigated whether the attenuated *S. typhimurium* strain CNC018 could similarly augment CRT exposure (Fig. 5A). In *s.c*. CT26 tumor-bearing BALB/c mice, *i.v*. administration of CNC018 markedly upregulated cell-surface CRT exposure within the tumor mass compared to PBS-treated controls (Fig. 5B). Consistently, *i.p.-*injected CRT3LFP and CRT3LP selectively localized to tumor tissues pretreated with CNC018, whereas the negative control monobody DGRLFP showed no such accumulation (Fig. 5C).

We next evaluated the therapeutic efficacy of these CRT-targeting proteins in combination with CNC018 (Fig. 5D-F). Monotherapy with CRT3LFP, CRT3LP, or DGRLFP yielded only marginal tumor suppression, which did not reach statistical significance. Furthermore, the combination of the control protein DGRLFP and CNC018 failed to enhance efficacy beyond that of CNC018 alone (Fig. 5D, E), indicating the therapeutic benefit is dependent on CRT-specific targeting. In contrast, the combination of CNC018 and CRT3LFP exhibited superior tumor suppression compared with CRT3LP, CRT3LFP, or CNC018 monotherapies (Fig. 5E). Notably, the synergistic combination of CNC018 + CRT3LFP was significantly more effective at tumor suppression than the CNC018 + CRT3LP combination (Fig. 5E, Fig. S3A, and Table S2), suggesting the addition of the FlaB component provides a distinct therapeutic advantage. This triple-component strategy (CNC018 + CRT3LFP) significantly prolonged mouse survival compared with all other experimental groups (Fig. 5F). Importantly, the treatment was well tolerated, with body-weight fluctuations remaining below 10% (Fig. 5G and Fig. S3B).

To confirm the generalizability of these findings, we extended our evaluation to C57BL/6 mice bearing *s.c*. MC38 tumors (Fig. 5H). Consistent with the CT26 model, the synergistic combination of CNC018 and CRT3LFP significantly suppressed tumor growth and improved survival rates compared with either agent alone (Fig. 5I, J, Fig. S4A, and Table S2) without compromising safety (Fig. S4B). Collectively, these results suggest that CRT3LFP robustly potentiates the antitumor activity of CNC018 across distinct genetic backgrounds, establishing it as a versatile platform for targeted cancer immunotherapy.

### Characteristics of immune profiling induced by CRT3LFP in CNC018-treated mice

**Fig. 6:**
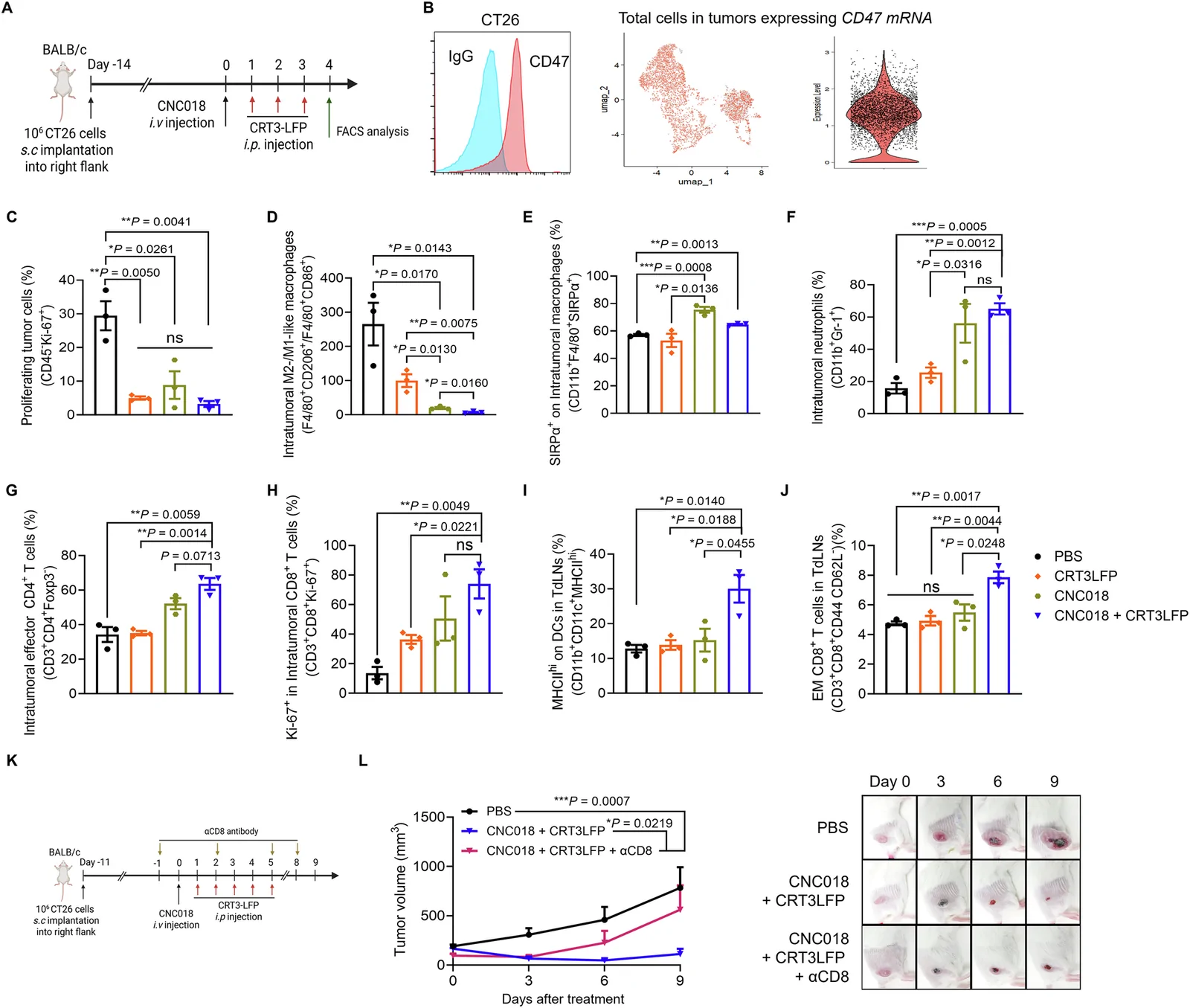
CRT3LFP enhances proinflammatory macrophage polarization and intra-tumoral immune cell infiltration in CNC018-treated mice. **A** Experimental scheme in CT26 tumor-bearing BALB/c mice co-treated with CNC018 and CRT3LFP (B-H). BALB/c mice with CT26 *s.c*. Tumor volumes of 120-140 mm³ were *i.v*. injected with CNC018 (2×10^7^ CFU) on day 0 (black arrow). CRT3LFP (4 IU/injection) *i.p*. administered three times from day 1 with 1-day interval (red arrows). Tumor tissues and TdLNs were obtained on day 4, and their cells were analyzed by flow cytometry (blue arrow). *P*-values were determined by an unpaired two-tailed *t*-test (*n* = 3 mice/group; ns, not significant). Figure created with BioRender.com. **B** Expression of CD47 in tumor tissues. BALB/c mice were implanted *s.c*. with CT26 cells (1 × 10^6^). When tumors reached ~120 mm³, tumor tissues were harvested for flow cytometry and single-cell RNA sequencing analysis. *Left*, flow cytometric analysis against CD47 on CT26 tumor cells. The cells were stained with specific antibodies (red). IgG, isotype controls (sky blue). Single-cell RNA sequencing analysis in total tumoral mononuclear cells. Middle, re-clustered UMAP plot of cells expressing *CD47* mRNA; right, violin plot of *CD47* mRNA level scores. mRNA of tumor tissues was isolated and pooled from 2 mice for analysis. **C** Frequency of proliferating tumor cells (CD45^-^Ki-67^+^) in tumor tissues. **D** Ratio of M2-like (F4/80⁺CD206⁺) to M1-like (F4/80⁺CD86⁺) macrophages in tumor tissues. **E** Frequency of SIRPα expression on intratumoral macrophages (CD11b⁺F4/80⁺SIRPα⁺). **F** Frequency of intratumoral neutrophils (CD11b⁺Gr-1⁺). **G** Frequency of intratumoral effector CD4⁺ T cells (CD3⁺CD4⁺Foxp3⁻). **H** Frequency of intratumoral activated CD8⁺ T cells (Ki-67⁺CD3⁺CD8⁺). **I** Frequency of activated DCs (CD11b⁺CD11c⁺MHCII^hi^) in TdLNs. **J** Frequency of effector memory (EM) CD8⁺ T cells in TdLNs (CD3⁺CD8⁺CD44⁺CD62L⁻). **K** Experimental scheme of CD8⁺ T cell depletion in CT26 tumor-bearing BALB/c mice co-treated with CNC018 and CRT3LFP CT26 *s.c*. tumor-bearing mice were *i.v*. injected with CNC018 (2 × 10^7^ CFU) on day 0 and subsequently *i.p*. injected five times with CRT3LFP (4 IU/injection) from day 1 with a 1-day interval. Anti-CD8 (αCD8) antibody (200 µg/injection) was *i.p*. injected three times to deplete CD8⁺ T cells. Figure created with BioRender.com. **L** Average tumor growth curves (left) and representative tumor images (right) of CT26 tumor-bearing BALB/c mice treated with CNC018 + CRT3LFP after CD8⁺ T cell depletion (*n* = 3 mice/group; two-way ANOVA with Tukey’s multiple comparisons test).

To examine whether CRT3LFP additively enhances antitumor effects in mice pretreated with CNC018, we analyzed the immune profiles of CT26 tumor tissues and tumor-draining lymph nodes (TdLNs) at 4 days post-injection (dpi) (Fig. 6A). We first confirmed the expression level of the targetable immune checkpoint CD47 on CT26 tumor cells. Flow cytometry showed robust CD47 surface expression compared to isotype control (Fig. 6B, left), which is consistent with high levels of *Cd47* mRNA within the tumor mass (Fig. 6B, right). All treatments (CNC018, CRT3LFP, and CNC018 + CRT3LFP combination) significantly decreased tumor cell proliferation (CD45^-^Ki-67^+^) compared to the PBS control (Fig. 6C). Although CRT3LFP monotherapy was less potent than CNC018 alone, it significantly shifted the macrophage polarization by decreasing the M2-like (F4/80⁺CD206⁺) to M1-like (F4/80⁺CD86⁺) ratio (Fig. 6D and Fig. S5). The combination of CNC018 and CRT3LFP further decreased this ratio compared with CNC018 monotherapy, indicating that CRT3LFP reshapes the proinflammatory polarization induced by CNC018. Consistent with this shift, CRT3LFP treatment, particularly in combination with CNC018, significantly downregulated the expression of the inhibitory receptor SIRPα on macrophages (CD11b⁺F4/80⁺SIRPα⁺) (Fig. 6E and Fig. S5). In contrast, while CNC018 increased intratumoral neutrophil frequencies (CD11b⁺Gr-1⁺), CRT3LFP did not significantly alter these populations, even in the combination group (Fig. 6F).

The innate immune reprogramming was accompanied by a robust recruitment of adaptive immune cells. The combination of CNC018 and CRT3LFP significantly increased the frequencies of intratumoral effector CD4⁺ T cells (CD3⁺CD4⁺Foxp3⁻) (Fig. 6G and Fig. S5) and proliferating CD8⁺ T cells (CD3⁺CD8⁺Ki-67⁺) (Fig. 6H and Fig. S5). Furthermore, in the TdLNs, we observed an enrichment of activated dendritic cells (DCs; CD11b⁺CD11c⁺MHCII^hi^) and effector memory (EM) CD8⁺ T cells (CD3⁺CD8⁺CD44⁺CD62L⁻) (Fig. 6I, J and Fig. S5). These data suggest that CRT3LFP additively enhances both innate sensing and adaptive antitumor immunity initiated by CNC018.

To determine whether the therapeutic efficacy of the CNC018 + CRT3LFP combination was dependent on the adaptive immunity, we performed a CD8⁺ T cell depletion study using an anti-CD8 neutralizing antibody (αCD8) (Fig. 6K). The loss of CD8⁺ T cells almost entirely abrogated the antitumor effects of the combination therapy (Fig. 6L and Fig. S6), indicating that CD8⁺ T cells are indispensable for the observed synergy. These findings suggest that while CNC018 initiates a modest immune response, the addition of CRT3LFP synergistically reprograms the TME by modulating macrophage polarization and driving a robust, T cell-dependent antitumor response.

### Antitumor immune efficacy of CRT3LFP and αCD47 Ab in CNC018-treated mice

**Fig. 7:**
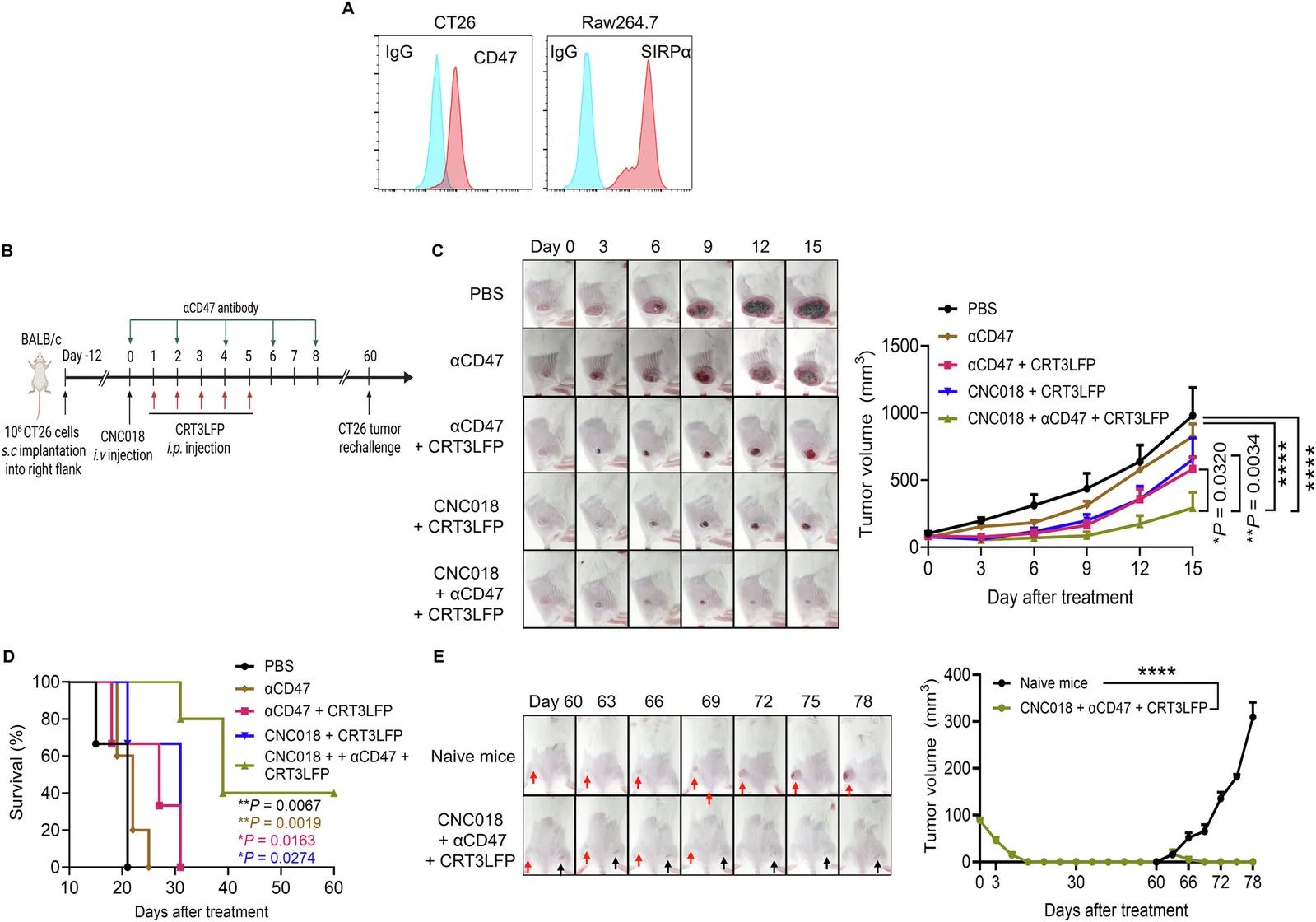
CD47 blockade enhances the antitumor therapeutic effect and immune memory in mice co-treated with CNC018 and CRT3LFP. **A** Expression of CD47 and SIRPα on tumor and macrophage cell lines. CT26 tumor and RAW264.7 macrophage cells were cultured in vitro, stained with αCD47 Ab (left) and anti-SIRPα (right) antibodies, respectively, and analyzed with flow cytometry (coral colors). IgG, isotype controls (sky blue). **B** Experimental scheme of CD47 blockade in CT26 tumor-bearing BALB/c mice co-treated with CNC018 and CRT3LFP (**C**, **D**). Mice with tumors (100-120 mm^3^) received CNC018 (2 × 10^7^ CFU, day 0, *i.v*. injection) and/or CRT3LFP (4 IU/injection, five times, *i.p*. injections with 1-day intervals from day 1). An αCD47 Ab (200 µg/injection) was administered *i.p*. five times with a 2-day interval from day 0. For tumor rechallenge, cured mice were rechallenged with CT26 cells (1 × 10^6^, *s.c*.) on the contralateral flank on day 60. Age-matched naive BALB/c mice were *s.c*. inoculated with CT26 cells as a control. Figure created with BioRender.com**. C** Representative images (left) and average growth curves (right) of treated CT26 tumors (PBS, αCD47 Ab + CRT3LFP, CNC018 + CRT3LFP, *n* = 6 mice/group; αCD47 Ab, *n* = 5 mice/group; CNC018 + αCD47 Ab + CRT3LFP, *n* = 8 mice/group; ****P < 0.0001; ns, not significant; two-way ANOVA with Tukey’s multiple comparisons test from two independent experimental replicates). **D** Kaplan-Meier survival curves for CT26 tumor-bearing mice from (**C**) [PBS, αCD47 Ab + CRT3LFP, CNC018 + CRT3LFP, *n* = 3 mice/group; αCD47 Ab, *n* = 5 mice/group; CNC018 + αCD47 Ab + CRT3LFP, *n* = 5 mice/group; Log-rank (Mantel-Cox) test]. **E** Representative images (left) and average growth curves (right) following CT26 tumor rechallenge (naive mice, *n* = 3 mice/group; cured mice, *n* = 2 mice/group; ****P < 0.0001; ns, not significant; two-way ANOVA with Tukey’s multiple comparisons test). Scars of primary tumors and rechallenge sites are marked by black and red arrows, respectively.

Macrophage-mediated phagocytosis is a critical component of innate tumor suppression, yet it is often subverted by the CD47-SIRPα “don’t eat me” signaling axis. Although antibodies targeting this checkpoint have shown promise in multiple preclinical and clinical trials as antitumor agents, their efficacy as monotherapies is often limited [37]. Given our observation that CNC018 increases SIRPα expression on macrophages and that CRT3LFP may partially mitigate this effect (Fig. 6E). Consistently, we hypothesized that a blockade of the CD47-SIRPα axis between macrophages and tumor cells in the TME would maximize the phagocytic potential of the CNC018 + CRT3LFP platform. We first confirmed high surface expression of CD47 on CT26 cells and SIRPα on RAW264.7 macrophages in vitro (Fig. 7A), consistent with our in vivo findings (Fig. 6B, right and E).

We next evaluated the therapeutic effects of an αCD47 Ab alone or incorporating it with CRT3LFP (Fig. 7B). The αCD47 monotherapy showed no significant antitumor activity compared with the PBS group, indicating that robust tumor control and survival benefit required the synergistic combination with CNC018 and CRT3LFP (Fig. 7C, D). While the αCD47 + CRT3LFP combination markedly inhibited tumor growth, the triple combination (CNC018 + αCD47 + CRT3LFP) showed the most robust antitumor activity (Fig. 7C and Fig. S7). This triple-component therapy induced complete tumor eradication in 40% (2 of 5) of the treated mice by day 12 (Fig. 7D). To determine the underlying mechanism, specifically whether CRT3LFP and CNC018 modulate macrophage polarization against tumors, we treated RAW264.7 cells with CRT3LFP, CNC018, or their combination and analyzed by flow cytometry for CD206 (M2-like macrophage) and CD86 (M1-like macrophage). Treatment with either CRT3LFP or CNC018 alone significantly reduced the CD206⁺/CD86⁺ (M2/M1) ratio compared to the PBS control. Notably, the combination of CRT3LFP and CNC018 further decreased this ratio relative to the CNC018 group, though no significant difference was observed compared with the CRT3LFP group, likely due to a saturated stimulatory effect of CRT3LFP on macrophage polarization (Fig. S8). These results suggest that CRT3LFP and CNC018 facilitate macrophage reprogramming toward a pro-inflammatory M1-like phenotype, which likely contributes to the enhanced antitumor efficacy observed in the CT26 tumor model.

To determine whether this combination therapy established durable immunological memory, we rechallenged the surviving tumor-free mice with a second injection of CT26 cells on the contralateral flank on day 60. While age-matched naive controls rapidly developed tumors, all previously cured mice remained completely resistant to the rechallenge (Fig. 7E). These findings indicate that the integration of αCD47 immunotherapy potentiates the tumor-suppressive effects of CNC018 and CRT3LFP and induces a potent and lasting systemic antitumor vaccination effect. This suggests a multifaceted mechanism in which the enhancement of macrophage-mediated phagocytosis and subsequent antigen presentation orchestrates a robust adaptive immune memory response.

## Discussion

L-ASNase is a standard clinical treatment for leukemia. However, it has largely failed in clinical trials for solid tumors because of the robust expression of asparagine synthetase (ASNS), which allows solid tumors to bypass systemic depletion through de novo biosynthesis [29, 38]. Unlike auxotrophic amino-acid-depleting agents such as arginine deiminase [39], the efficacy of L-ASNase is strictly dependent on the tumor’s metabolic flexibility. Furthermore, the clinical utility of native L-ASNase is severely hampered by off-target toxicity toward hematopoietic progenitors and hepatocytes, which rely on extracellular asparagine for rapid protein synthesis, often resulting in dose-limiting lymphopenia and coagulopathy [40, 41]. Recent studies have demonstrated that by tethering L-ASNase to a CRT-targeting monobody, metabolic stress can be concentrated within the TME, thereby expanding the therapeutic window and mitigating systemic liabilities.

CRT-targeting L-ASNase has shown promise in sensitizing tumors to radiotherapy and chemotherapy through the induction of ICD [30, 31]. However, it fails to generate curative immunity, as the compensatory upregulation of PD-L1 and the expansion of immunosuppressive TAMs collectively dampen T-cell-mediated responses [8, 9, 30, 33]. Specifically, radiotherapy is inherently limited by spatial constraints and poor efficacy in the hypoxic and necrotic cores of advanced solid tumors [30]. By contrast, bacteriotherapy with tumor-colonizing strains, including attenuated *Salmonella*, can selectively target hypoxic tumor niches, reprogram the immunosuppressive TME, and induce durable systemic antitumor immunity [13, 14, 16, 18, 20, 21, 24]. We show that CNC018 acts as a biological “primer”, inducing surface CRT exposure and providing a high-density target of CRT3LFP. This bacterial-metabolic axis overcomes the spatial heterogeneity of conventional treatments and establishes a foundational platform for comprehensive TME remodeling.

Here, we developed CRT3LFP to couple metabolic deprivation with innate immune activation. By integrating the TLR5-agonist FlaB, we achieved a coordinated reprogramming of the TME. While L-ASNase activity inhibits tumor cell proliferation and is consistent with recent reports that potentiate CD8^+^ T-cell effector function [27], FlaB facilitates the reprogramming of TAMs from an M2 to an M1-like phenotype. In combination with CNC018, CRT3LFP facilitates comprehensive TME reprogramming, characterized by increased activated DCs, effector CD4^+^ T cells, and effector memory CD8^+^ T cells. However, we observed that reprogramming TAMs was insufficient for complete tumor regression, suggesting the existence of predominant immune-evasion pathways.

The CD47-mediated “don’t-eat-me” signal serves as a potent antagonist to the ‘eat-me’ signal (CRT) induced by CNC018, thereby limiting macrophage-mediated phagocytosis. By incorporating CD47 blockade, CRT3LFP effectively reshapes TAMs toward a pro-phagocytic phenotype. This triple combination of CNC018, CRT3LFP and αCD47 Ab inhibited tumor growth and established durable immunological memory, enabling mice to resist subsequent tumor rechallenge. The successful rejection of tumor rechallenges in mice pretreated with the CNC018-based triple-combination regimen suggests the induction of durable antitumor immunity. However, because αCD47 Ab was included during the initial treatment phase, this effect cannot be attributed solely to CNC018 + CRT3LFP. Previous studies have shown that CD47 blockade can promote dendritic cell-mediated cross-priming of CD8⁺ T cells and contribute to durable antitumor memory [42]. In parallel, *Salmonella*-based cancer therapy has been reported to induce immunogenic cancer cell death, enhance dendritic cell maturation, and promote tumor-specific memory T-cell responses [23, 43]. Therefore, our findings suggest that the observed protection against tumor rechallenge may arise from the combined immunological effects of CNC018, CRT3LFP, and αCD47 Ab, rather than from CNC018 + CRT3LFP alone. The specific contribution of αCD47 Ab in combination with CNC018 + CRT3LFP to enhanced antitumor immune memory remains to be defined in future studies. Nonetheless, our results highlight the potential of a tripartite approach that integrates metabolic stress (via L-ASNase) and innate immune activation (via FlaB) with the neutralization of phagocytic checkpoints, thereby optimizing the efficacy of the ‘eat-me’ signaling initiated by CNC018-induced CRT exposure.

Despite these promising results, several challenges persist regarding clinical translation. The metabolic vulnerability of malignancies to CRT3LFP is likely conditioned by heterogeneous expression of asparagine synthetase and CD47 across diverse tumor types, including pancreatic cancer and glioblastoma. Additionally, the inherent immunogenicity of bacterially derived enzymes often precipitates the development of neutralizing antibodies and hypersensitivity reactions [44]. To mitigate these liabilities, we employed PASylation, the genetic incorporation of proline-, alanine-, and serine-rich polypeptides, to shield immunogenic epitopes and prolong the circulating half-life of the therapeutic. Building upon our previous validation of this strategy for CRT-targeting agents, we demonstrate its necessity for sustaining a robust metabolic–immune response in vivo [31]. Collectively, our findings establish a compelling foundation for a ‘bacterial-metabolic-checkpoint’ paradigm, providing a versatile and potent framework to circumvent the multifaceted resistance mechanisms inherent to solid tumors.

## Materials and methods

### Bacterial strains, cancer cell lines, and reagents

*S. typhimurium* CNC018 strain (Δ*relA*Δ*spoT*ΔSPI-1ΔSPI-2) is an attenuated strain in which two genes of ppGpp biosynthesis (*relA* and *spoT*) and two virulence gene clusters of type III secretion systems (T3SSs) responsible for host invasion and intracellular survival, respectively, were disrupted via homologous recombination [23–25].

Murine colorectal carcinoma cells CT26 (CRL-2638, ATCC, USA) and murine colon adenocarcinoma cells MC38 (ENH204-FP, Kerafast, USA); murine macrophage cells RAW264.7; and the HEK293/mTLR5 cell line, which is a human embryonic kidney 293 (HEK293) cell stably transfected with the murine TLR5 (mTLR5) gene (Cat. Code: 293-htlr5, InvivoGen, Hong Kong), were used in this study. All cells were cultured in high-glucose Dulbecco’s Modified Eagle’s Medium (DMEM) (LM-001-05, Welgene, Korea) supplemented with 10% fetal bovine serum (FBS) (S101-07, Welgene, Korea) and 1% penicillin-streptomycin (LS203-01, Welgene, Korea) at 37 °C in a 5% CO_2_ atmosphere and routinely evaluated for mycoplasma contamination using a PCR-based detection kit (25237, Intron Biotechnology, Korea) before experiments.

The L-ASNase activity assay kit (ab107922) and recombinant CRT (rCRT) were obtained from Abcam, USA. Recombinant *E. coli* L-ASNase and Cell Counting Kit-8 (CCK-8) were obtained from Prospec, USA (ENZ-287), and from Enzo Life Sciences, USA (ALX-850-039-KI01), respectively.

All antibodies used in this study are listed in Table S1.

### Generation of expression plasmids for CRT3LFP and its control DGRLFP

pETh-CRT3-LASP-PAS200 and pETh-Fn3(DGR)-LASP-PAS200 were the expression plasmids for CRT3LP and DGRLP, PASylated (P) L-ASNases (L) with CRT-targeting monobody (CRT3) or its control [Fn3(DGR); DGR], respectively, and pBS-BglII-G4S-PAS200-BamHI was a plasmid containing the PAS200 sequence and previously described [31].

To generate PASylated FlaB genes, we amplified a *flaB* gene fragment with primers EcoRI-FlaB-F (5’-GATCGAATTCGCAGTGAATGTAAATACAAACGTAGCAGC) and BamHI-FlaB-R (5’-GATCGGATCCGCCTAGTAGACTTAGCGCTGAGTTTGGCGC) against a template plasmid pJH18-FC using polymerase chain reaction (PCR). The fragment was digested with *Eco*RI and *Bam*HI and ligated into the *Eco*RI and *Bgl*II sites of pBS-BglII-G4S-PAS200-BamHI, named pBS-FlaB-PAS200. The 1.8-kb FlaB-PAS200 fragment was purified from this plasmid after digestion with *Eco*RI and *Bam*HI. Fragments CRT3-LASP and Fn3(DGR)-LASP were amplified with primers T7 and LASP-G4S-MfeI-R (5’-CACTGCCAATTGGCTGCCGCCGCCGCCGCTGCCGCCGCCGCCGCTGTACTGATTGAAGATCTGCTGGATC) against plasmids pETh-CRT3-LASP-PAS200 and pETh-Fn3(DGR)-LASP-PAS200, respectively, and digested with *Nhe*I and *Eco*RI. These digested fragments and FlaB-PAS200 digested with *Eco*RI and *Bam*HI were triply ligated into the *Nhe*I and *Bam*HI sites of the pETh vector and named pETh-CRT3LFP and pETh-Fn3(DGR)LFP, respectively. As a result, the genes of monobody, L-ASNase, FlaB, and PAS200 tag were linked via (G4S)_2_ linkers, and a His-tag was added at their C-termini.

All PCR reactions were performed using speed-*pfu* polymerase (S250N, Nahelix, Korea), and restriction enzymes were obtained from New England Biolabs, USA. The sequences of all plasmids were confirmed by Macrogen, Korea.

### Recombinant protein purification

*E. coli* BL21(DE3) competent cells (CP110, Enzynomics, Korea) were transformed with expression vectors and cultured overnight at 37 °C in LB broth supplemented with 50 µg/mL kanamycin. The next day, the cultures were diluted to a 1:50 ratio into 500 mL of fresh LB broth containing kanamycin and further incubated at 37 °C for 3 h. Protein expression was induced with 0.5 mM isopropyl-β-D-1-thiogalactopyranoside (IPTG; Sigma-Aldrich, USA), followed by incubation at 25 °C for 24 h. Bacterial cells were harvested by centrifugation at 6000 × *g* for 10 min at 4 °C.

Cell pellets were lysed by sonication, and the expressed proteins were purified using a HisTrap HP 5 mL column (11003399, Cytiva, USA). The column was washed with buffer (50 mM Tris, pH 7.4; 500 mM NaCl; 40 mM imidazole; 0.5 mM dithiothreitol, DTT) and eluted with buffer containing 500 mM imidazole in 50 mM Tris (pH 7.4) and 500 mM NaCl. Protein samples were buffer-exchanged into PBS using PD-10 desalting columns (17-0851-01, Cytiva, USA). The purified proteins were treated with high-capacity endotoxin removal spin columns (Thermo Fisher Scientific, USA) to remove bacterial endotoxin and stored in PBS at −20 °C.

### Sodium dodecyl sulfate-polyacrylamide gel electrophoresis (SDS-PAGE) and Western blotting analysis

The purified proteins were blended with a 2X sample buffer (EBA-1052, Elpis Biotech, Korea), then boiled at 95 °C for 10 min and loaded onto 10% SDS-PAGE gels (20 μg per well). After electrophoresis, the proteins were stained with Coomassie blue R-250 staining solution (EBC001-500, Enzynomics, Korea).

To assess the expression level and purity of recombinant proteins, Western blot analysis was performed with specific antibodies. Briefly, proteins on SDS-PAGE gels were transferred to nitrocellulose membranes at 110 V for 90 min. The membranes were blocked in Tris-buffered saline with 1% Tween-20 (TBS-T; T0161CD, BYLABS, Korea) containing 5% skim milk overnight at 4 °C. After removing excess blocking solution, the membranes were incubated with a rabbit anti-His-tag monoclonal antibody (1:1000 dilution) for 2 h at room temperature, followed by five washes with TBS-T. Subsequently, membranes were probed with a horseradish peroxidase (HRP)-conjugated anti-rabbit secondary antibody (1:4000 dilution) for 1 h. After five-time TBS-T washing, a chemiluminescent HRP substrate (WBKLS0700, Merck Millipore, USA) was added. The specific protein bands were detected using the ChemiDoc^TM^ XRS+ system imager (Bio-Rad, USA).

The L-ASNase and FlaB moieties of CRT3LFP and DGRLFP were confirmed with an anti-L-ASNase polyclonal antibody (ab55824, Abcam, USA) and an anti-FlaB polyclonal antibody (Lab stock) as the primary antibodies using the same method described above.

### L-ASNase activity assay

L-ASNase activity was assessed following the method of Wriston and Yellin [45], which measures ammonia liberation via nesslerization. The reaction mixture comprised 1.5 mL of 0.04 M L-asparagine in 0.05 M Tris-HCl buffer (pH 8.6) and 0.5 mL of enzyme solution, resulting in a total volume of 2 mL. This mixture was incubated at 37 °C for 30 min, and the reaction was terminated by adding 0.5 mL of 1.5 M trichloroacetic acid (TCA). A control was prepared by adding TCA to the enzyme solution prior to mixing with the substrate. Post-reaction, the mixture was centrifuged at 10,000 × *g* for 15 min to remove precipitated proteins, and the supernatant was collected. For ammonia quantification, 0.5 mL of supernatant was diluted to 7 mL with distilled water, followed by the addition of 1 mL of Nessler’s reagent. After 20 min, OD480 was measured. Ammonia concentration was determined using a standard curve established with ammonium sulfate solutions. One international unit (IU) of L-ASNase activity is defined as the enzyme quantity catalyzing the release of 1 μmol of ammonia per minute at 37 °C and pH 8.6.

### Measurement of binding affinity using enzyme-linked immunosorbent assay (ELISA)

The binding affinity of CRT3LFP against CRT was assessed as previously described [46]. Briefly, 100 μL of CRT3LFP (0**-**10 μM/well) was added in each well of a 96-well microplate. After overnight incubation at 4 °C, unbound proteins were removed by aspiration. Subsequently, 100 μL of 10 μM rCRT was added to each well and incubated for 2 h at room temperature. After removing unbound rCRT, rabbit anti-CRT antibody (1:500 dilution) was added and incubated for 2 h at room temperature. The antibodies were aspirated, and wells were washed five times with PBS containing 1% Tween-20 (T-PBS; T9181, Takara, Japan). Biotin-conjugated anti-rabbit secondary antibody (1:1000 dilution) was then added and incubated for 1 h. Following five additional T-PBS washes, 100 μL of avidin-HRP (1:250 dilution) was added to each well and incubated for 30 min at room temperature. After aspiration and five further washes, 100 μL of 1× 3,3′,5,5′-tetramethylbenzidine (TMB) substrate (Thermo Fisher Scientific, USA) was added to generate a yellow reaction product. The reaction was terminated after 15 min by adding 50 μL of 0.5 M H₂SO₄ per well. The optical density at 450 nm (OD450) was measured using a SpectraMax M2 microtiter plate reader (Molecular Devices, USA) to quantify the colorimetric product.

### RAW264.7 macrophage polarization assay

The RAW264.7 macrophage cell line (1 × 10^6^ cells/well) was seeded in 12-well plates and allowed to adhere overnight. CNC018 bacteria were grown in LB broth to approximately 2 OD600, and the supernatant was collected by centrifugation and passed through a 0.2-µm sterile filter to remove residual bacteria. 5 µL of the filtered supernatant (20 µg total protein) was added to each well of RAW264.7 cells. In parallel, the cells were treated with CRT3LFP at a final concentration of 4 IU/well, alone or in combination with the CNC018 supernatant. After 24 h of incubation, the cells were detached from the culture plates and stained with FITC-conjugated anti-CD86 and PE-conjugated anti-CD206 antibodies for flow cytometry analysis to quantify M1- and M2-like macrophage populations.

### Establishment of tumor animal models

Six-week-old female BALB/c and C57BL/6 mice, weighing approximately 19 g, were obtained from Samtako, Korea. The animals were acclimatized for one week in a specific pathogen-free facility. Mice were randomly selected from different cages and cohoused in new cages according to experimental groups, with each mouse used in only one experiment. All procedures adhered to the guidelines of the Chonnam National University Animal Experimental and Ethics Committee (Ethics Approval No. CNU IACUC-H-2026-3) and the principles of the National Centre for Replacement, Refinement, and Reduction of Animals in Research [14, 47].

CT26 (1 × 10^6^) or MC38 (1 × 10^6^) tumor cells in 50 µL PBS were subcutaneously (*s.c*.) implanted into the flank of BALB/c or C57BL/6, respectively, under 2% isoflurane anesthesia. After 10-11 days, when tumors reached 100–120 mm³, mice were treated with bacteria via the intravenous (*i.v*.) route and/or recombinant protein via the intraperitoneal (*i.p*.) route.

Tumor volume (mm³) was determined using the equation (length × width × height)/2. In accordance with the Chonnam National University Animal Experimental and Ethics Committee guidelines, mice with tumors reaching or exceeding 1500 mm³ were euthanized. In nearly all instances where this threshold was surpassed, the mice exceeded it for no more than 3 days. Mice exhibiting signs of pain, distress, discomfort, or over 10% of body weight loss were promptly euthanized. To reduce bias, all experiments and analyses were conducted in a blinded manner.

### Analyses of tumor-infiltrating immune cells

Solid tumor tissues and tumor-draining lymph nodes (TdLNs) were harvested from mice on day 4 post-bacterial treatment or after 3 time injections of recombinant protein. The samples were processed in 2 mL of isolation buffer (RPMI 1640 with 5% FBS, 1% L-glutamine, 1% penicillin-streptomycin, and 10 mM HEPES). After mechanical homogenization, the samples were incubated with 1 mg/mL collagenase type IV (Roche, Switzerland) and 50 μg/mL DNase I (Roche, Switzerland) for 45 min at 37 °C. Each 2-mL sample was mixed with an equal volume of 1× lysis buffer (BioLegend, USA) for 5 min at 37 °C to remove red blood cells. Samples were filtered through 100-μm and 40-μm cell strainers (BD Falcon, USA) and then incubated in Fc blocking buffer (101320, BioLegend, USA) for 15 min at room temperature. Cells were stained with specific fluorescent dye-conjugated antibodies following the manufacturer’s instructions (Table S1) for 30 min on ice. Fluorescence signals of the cells were analyzed using a FACS Canto II flow cytometer and FlowJo software_._ A minimum of 10^6^ cellular events was required to be recorded per sample.

### Single-cell RNA sequencing (scRNA-seq) analysis

The tumor tissues were isolated from CT26 tumor-bearing mice, and the cells were prepared as described above. The cell samples were sent to the sequencing facility (Macrogen, South Korea) for scRNA-seq as described previously [48].

Raw 10x Genomics count matrices were processed in R using Seurat (v5.0.0). Low-quality cells were removed by filtering on nFeature_RNA (500-8,000) and mitochondrial gene content ( < 5%), and putative doublets were identified and excluded with scDblFinder [49].

Each dataset was subjected to log-normalization, after which 2,000 highly variable genes were selected using the “vst” method. The data were subsequently scaled, and principal component analysis (PCA) was performed using the first 50 principal components. Batch effects were mitigated using Harmony (v0.1.1) [50]. Graph-based clustering was carried out with the Louvain algorithm over a resolution range of 0.2-1.2. For visualization, low-dimensional embeddings were generated by applying UMAP to the Harmony-corrected representations.

For CD47, per-sample expression was quantified as the proportion of cells with detectable transcripts (log-normalized expression > 0), and summary statistics and visualizations were produced using ggplot2 [51].

### Treatment of CNC018, CRT3LFP, and antibody in tumor-bearing mice

CT26 (1 × 10^6^) or MC38 (1 × 10^6^) tumor cells in 50 µL PBS were implanted *s.c*. into the flank of BALB/c or C57BL/6, respectively, under 2% isoflurane anesthesia. After 10–12 days, when tumors reached 100–140 mm³, mice received CNC018 (2 × 10^7^ CFU) treatment via *i.v*. injection (day 0). For CRT3LFP treatment, 45 µg (4 IU) in 200 µL PBS was injected *i.p*. starting one day after bacterial treatment, five times at 1-day intervals.

To neutralize CD8 T cells or to block CD47-SIRPα interaction, mouse anti-CD8 (BP0061, Bio X Cell, USA) or αCD47 (BE0270, Bio X Cell, USA) antibodies in 200 µL PBS (10 mg/kg body weight) were *i.p*. injected into CT26 tumor-bearing mice on day -1 or 0, respectively, and repeatedly injected as indicated. Rat IgG2b antibody (BE0090, Bio X Cell, USA) was used as an isotype control.

### Evaluation of the antitumor synergism by CNC018 and CRT3LFP combination therapy

The Coefficient of Drug Interaction (CDI) was calculated to assess whether the tumor suppression induced by the combination of CRT3LFP and CNC018 was synergistic, additive, or antagonistic, as previously described [17, 52, 53]. The CDI was determined using the formula CDI = (AB/control)/[(A/control) × (B/control)], where A and B represent the effects of the individual treatments (CNC018 alone and CRT3LFP alone), and AB denotes the effect of the combined treatment (CNC018 + CRT3LFP). CDI values < 1, =1, and >1 indicate synergism, additive effect, and antagonism, respectively. Data were derived from mean tumor volumes measured on day 15 for the CT26 tumor model or day 12 for the MC38 tumor model, as shown in Fig. 5E, I. The ratios of mean tumor volumes in the CNC018, CRT3LFP, and CNC018 + CRT3LFP groups, relative to the control group (PBS), were defined as “A”, “B”, and “AB”, respectively.

### Statistical analysis

Statistical analysis was conducted using GraphPad Prism 9.0 (GraphPad, USA), with significance set at *P* < 0.05. Specific statistical tests and *P* values are provided in figure legends. For single-variable comparisons, a student’s *t*-test or one-way ANOVA was applied. Two-way ANOVA with Tukey’s post-hoc correction was used for multiple comparisons, while survival analysis was performed using Kaplan-Meier curves with log-rank (Mantel-Cox) tests. All data are shown as mean ± standard error of the mean (s.e.m.).

## Supplementary information

Supplemental material

## Data Availability

All of the data reported in this study are available from the corresponding authors upon reasonable request.
