# Supplementary material for: Calreticulin-targeting L-asparaginase-flagellin conjugate enhances Salmonella-mediated antitumor efficacy
Source: Cell Death Discov. 2026 Aug 9;12:441. doi: 10.1038/s41420-026-03300-x (PMC13737104; doi:10.1038/s41420-026-03300-x)
Supplement: Supplementary file 1 [file 41420_2026_3300_MOESM1_ESM.doc]

Supplementary Materials for

**Calreticulin-targeting L-asparaginase-Flagellin conjugate enhances *Salmonella*-mediated antitumor efficacy**

Dinh-Huy Nguyen *et al.*

**Corresponding authors:**

Yeongjin Hong (yjhong@jnu.ac.kr), Jung-Joon Min (jjmin@jnu.ac.kr), and Wan-Sik Lee (iliad@jnu.ac.kr)

**This PDF file includes:**

Materials and Methods

Figures S1 to S8

Tables S1 to S2

**Materials and Methods**

**Measurement of binding affinity using surface plasmon resonance analysis**

Binding kinetics of CRT3LFP to CRT were determined by surface plasmon resonance using a Biacore X100 instrument (Cytiva, USA). rCRT was immobilized on a Series S Sensor Chip CM5 via amine coupling to a density of approximately 1,000-2,000 response units (RU). CRT3LFP was prepared in running buffer [10 mM HEPES (pH 7.4), 150 mM NaCl, 0.05% Tween-20] and injected as an analyte in a multi-cycle kinetic series at concentrations ranging from 0.3 to 130 nM. Association was monitored for 180-300 seconds, and dissociation for 600-1,200 seconds at a flow rate of 30-50 µL/min. The surface was regenerated with 10 mM glycine-HCl pH 2.0-2.5 (or the appropriate regeneration condition). Sensorgrams were fitted to a 1:1 Langmuir binding model using Biacore X100 Evaluation Software to derive the association rate constant (*k*_a_), dissociation rate constant (*k*_d_), and equilibrium dissociation constant (K_D_ = *k*_d_/*k*_a_).

**Protein stability assay in serum**

100 μL of CRT3LFP [60 μg or 8 IU in PBS (pH 7.4)] was mixed with the same volume of mouse blood serum in a low-binding 1.5-mL microcentrifuge tube and incubated at 37 °C. The aliquots (10 μL) were withdrawn at predefined time points and immediately cooled on ice. L-ASNase activity of each aliquot was determined as described above. The enzyme activity percentage = [(enzyme activity in CRT3LFP + serum) - (enzyme activity in serum alone) at the indicated time]/[(enzyme activity in CRT3LFP + serum) - (enzyme activity in serum alone) at t = 0] × 100. Recombinant L-ASNase was used as a positive control.

**FlaB activity assay**

HEK293/mTLR5 cells (3×10^4^) were plated in 96-well plates and incubated overnight at 37 °C. The cells were transiently transfected with 100 ng of pNiFty(2)-SEAP (Cat. code: 293-htlr5, InvivoGen, Hong Kong), a plasmid expressing a secreted embryonic alkaline phosphatase (SEAP) after activation of the mTLR5-NF-kB signaling pathway, in 10 μL TurboFectin reagent (Cat. code: TF81001, Origene, USA). After incubation for a day, the medium were replaced with 180 μL of fresh DMEM supplemented with 10% heat-inactivated FBS and then treated with 20 μL of 4 nM CRT3LFP. The purified FlaB was used as a positive control. After 6 h of incubation, 20 μL of culture supernatants were transferred into each well of a 96-well plate, and 180 μL of alkaline phosphatase detection reagent QUANTI-Blue™ (InvivoGen, USA) was added to each well. After 3 h, the OD620 value of each well was measured using a microplate reader to quantify the SEAP activity.

**Analysis of CD47 and SIRPα expressions on the surface of in vitro-cultured cells**

To assess the expression levels of CD47 on tumor cells and SIRPα on macrophages, tumor cells (CT26 and MC38) or macrophage cells (RAW264.7) were cultured in vitro. The overnight cultured cells (1×10^6^) were detached from the culture plates and stained with anti-CD47 antibody (10 μg/mL) against tumor cells or anti-SIRPα antibody (12.5 μg/mL) against macrophages, respectively, on ice for 30 min. The stained cells were washed by FACS buffer and then fixed in 1% PFA. The corresponding PE-conjugated isotype antibodies (IgG) were used as controls.

Fluorescence levels of the stained cells were measured with a FACSCanto II Flow Cytometer and analyzed using FlowJo software. A minimum of 10,000 events were recorded per sample.

**In vitro binding assay of CRT3LFP against the tumor cells treated with CNC018**

CT26 or MC38 cells (2×10^5^ cells) were cultured overnight in DMEM supplemented with 10% FBS at 37 °C in a 5% CO_2_ atmosphere in 6-well plates. The medium was removed by aspiration, and fresh DMEM without antibiotics containing CNC018 (2×10^6^ CFU/mL) was added to each well. The cells were incubated for 22 h, followed by a 2 h treatment with penicillin-streptomycin (LS203-01, Welgene, Korea) to a final 1% concentration. Then, the cells were detached using 0.05% trypsin-EDTA (LS-015-01, Welgene, Korea) and washed three times with FACS buffer (1% FBS in PBS; OPBDPWO50, Welgene, Korea). To assess CRT exposed on the cell surface, cells were treated with 50-fold diluted PE-conjugated anti-CRT antibody (19780S, Cell Signaling, USA) for 30 min and incubated on ice. The same cells were treated with 100 nM CRT3LFP as well as DGRLFP and CRT3LP for 1 h on ice. All the treated cells were washed five times with FACS buffer and treated with an anti-His tag monoclonal antibody (1:1,000 dilution, ab245114, Abcam, USA) for 1 h. After simple washing, the cells were treated with 5 μg/mL Alexa Fluor 488-conjugated secondary antibody (A11008, Thermo Scientific, USA) for 1 h. Fluorescence signals of the cells were analyzed using a FACS Canto II flow cytometer (BD Biosciences, USA) and FlowJo software (Becton, Dickinson & Company, USA).

**Confocal immunofluorescence imaging analysis**

CRT3LFP, as well as DGRLFP and CRT3LP bound to CRT exposed on cell membranes, were visualized as previously described using confocal immunofluorescence microscopy^6,31,32^. CT26 and MC38 cells (1×10^4^) were cultured overnight on cover glass and treated with CNC018 (1×10^5^) at 37 °C for 22 h. Thereafter, the cells were fixed with 1% paraformaldehyde (PFA; Biosesang, South Korea) for 15 min and then sequentially stained with anti-CRT antibody (1:1000 dilution) on ice for 1 h and Alexa Fluor 488-conjugated secondary antibody (5 μg/mL) for 1 h. The same processed cells were incubated with 100 nM recombinant proteins on ice for 1 h and then sequentially stained with anti-His tag monoclonal antibody (1:1,000 dilution) for 1 h and Alexa Fluor 488-conjugated secondary antibody (5 μg/mL) for 1 h.

Cell membranes were labeled with Alexa Fluor 555-conjugated wheat germ agglutinin (WGA) (1:5,000 dilution; Thermo Fisher Scientific, USA). Samples were washed with cold 1% Tween-PBS between each step. Nuclei were stained with 4′,6-diamidino-2-phenylindole (DAPI) (Cat# P36931, Thermo Fisher Scientific, USA). The staining with anti-CRT antibody (1:1000 dilution) was also done using sequential staining with anti-CRT and Alexa Fluor 488-conjugated secondary antibodies.

Imaging was performed using an LSM510 confocal microscope (ZEISS, Jena, Germany), and data were analyzed with ZEN-LSM imaging software (ZEISS, Jena, Germany).

**Cell viability assay**

CT26 and MC38 cells (1×10^4^) were plated in triplicate in a 96-well plate and cultured at 37 °C. The next day, the culture medium was replaced by 100 µL of fresh media containing CNC018 bacteria (1×10^5^ CFU). After 18 h of culture, penicillin-streptomycin was added to each well to achieve a final concentration of 1%, and the culture continued for an additional 2 h. The PBS-washed cells were treated with 5 IU/mL of CRT3LFP as well as CRT3LP and DGRLFP and cultured for 4 h at 37 °C. Then, CCK-8 reagent (10 μL) was added to each well and incubated at 37 °C for 1-2 h. The OD_450_ of each well was measured to evaluate the viable cell level by a SpectraMax M2 microtiter plate reader (Molecular Devices, USA).

**In vivo toxicity assay of CRT3LFP in mice**

Female 7-week-old BALB/c mice (Samtako, Korea) were implanted subcutaneously (*s.c.*) with CT26 cells (5×10^5^) in the right flank. When the tumor volumes reached approximately 100-120 mm^3^, CRT3LFP (4, 8, or 12 IU) was *i.p.* injected into mice. PBS was used as an untreated control. Body weight, tumor size, and survival of mice were observed at the indicated days.

**Supplementary figures**

**Figure S1. The binding affinity of CRT3LFP to calreticulin (CRT) is determined by surface plasmon resonance (SPR).** Sensorgrams showing the real-time binding of CRT3LFP (analyte) at various concentrations (0.3 nM, blue; 1.5 nM, red; 15 nM, green; 40 nM, purple; 80 nM, orange; 130 nM, black) to immobilized CRT (ligand). The curves display association and dissociation phases, with global fitting to a 1:1 binding model yielding an equilibrium dissociation constant (K_D_) of 4.52 × 10^−9^ M, an association rate constant (*k*_a_) of 8.51 × 10^4^ M^−1^s^−1^, and a dissociation rate constant (*k*_d_) of 3.85 × 10^−4^ s^−1^.

**Figure S2. Evaluation of tumor growths and body weight changes in individual CT26 tumor-bearing mice treated with CRT3LFP** (related to Figure 4, *n*=3 mice/group).
(**A**) Tumor growth curves in individual mice treated with PBS or CRT3LFP (4 IU, 8 IU, or 12 IU).
(**B**) Body weight changes in individual mice treated with PBS or CRT3LFP (4 IU, 8 IU, or 12 IU).

**Figure S3. Therapeutic effects of CNC018 and CRT3LFP co-treated in individual CT26 tumor-bearing mice** (related to Figure 5D-G). (**A**) Individual tumor growth curves in CT26 tumor-bearing mice co-treated with the indicated treatments. CNC018 (2×10^7^ CFU) was *i.v.* administered on day 0. The proteins (CRT3LP, DGRLFP, and CRT3LFP; 4 IU/injection) were *i.p.* given five times with a 1-day interval from day 1. PBS was treated as a control (PBS, CRT3LP, DGRLFP, CRT3LFP, CNC018, CNC018 + CRT3LP, CNC018 + CRT3LFP, *n*=4 mice/group; CNC018 + DGRLFP, *n*=3 mice/group). (**B**) Body weight changes in individual mice across treatment groups listed in (A).

**Figure S4. Therapeutic effects of CNC018 and CRT3LFP co-treated in individual MC38 tumor-bearing mice** (related to Figure 5H, I). CNC018 (2×10^7^ CFU) was *i.v.* administered on day 0. CRT3LFP (4 IU/injection) was *i.p.* given five times with a 1-day interval from day 1 (PBS, *n*=3 mice/group; CRT3LFP, CNC018, CNC018 + CRT3LFP, *n*=4 mice/group). (**A**) Individual tumor growth curves in MC38 tumor-bearing mice. (**B**) Individual body weight changes in mice of (A).

**Figure S5. Gating strategies of flow cytometry analysis in CT26 tumor tissues and tumor-draining lymph nodes (TdLNs)** (related to Figure 6A-J). After harvesting cells in tumor tissues and TdLNs, they were stained with a Live/Dead cell kit, anti-CD45 (leukocyte marker), anti-CD16/CD32 (to block non-specific Fc receptor binding), and additional immune cell-specific antibodies. (**A**) Gating strategy for tumor tissue analysis. (**B**) Gating strategy for TdLN analysis.

**Figure S6. CD8^+^ T cell depletion effect in individual CT26 tumor-bearing BALB/c mice co-treated with CNC018 and CRT3LFP** (related to Figure 6L). CT26 *s.c.* tumor-bearing BALB/c mice (*n*=3) were *i.v.* treated with CNC018 (2×10^7^ CFU) on day 0, and subsequently *i.p.* treated with CRT3LFP (4 IU/injection) five times from day 1 with a 1-day interval. The anti-CD8 (αCD8) antibody (200 µg/injection) was *i.p.* injected three times with a 3-4-day interval. Individual tumor growth curves are shown.

**Figure S7. CD47 blockade enhances antitumor therapeutic effect in mice co-treated with CNC018 and CRT3LFP** (related to Figure 7C). Mice bearing CT26 tumors (100-120 mm^3^ (PBS, αCD47 + CRT3LFP, CNC018 + CRT3LFP, *n*=6 mice/group; αCD47, *n*=5 mice/group; CNC018 + αCD47 + CRT3LFP, *n*=8 mice/group) received CNC018 (2×10^7^ CFU, day 0, *i.v.* injection) and/or CRT3LFP (4 IU, five-time *i.p.* injections with a 1-day interval from day 1). αCD47 antibody (200 µg/injection) was administered *i.p.* five times with a 2-day interval from day 0. Individual tumor growth curves of CT26 tumor-bearing mice are shown.

**Figure S8. CRT3LFP and CNC018 promote macrophage polarization toward an M1-like phenotype.**
RAW264.7 cells were treated with PBS, CRT3LFP (4 IU), CNC018, or CNC018 + CRT3LFP (4 IU), and the proportion of M2-like/M1-like macrophages was analyzed by flow cytometry based on CD206 and CD86 expression. CRT3LFP or CNC018 alone significantly reduced the proportion of CD206⁺/CD86⁺ M2/M1-like macrophages compared with PBS control. The combination of CRT3LFP and CNC018 further decreased this ratio compared with the CNC018 group, with no significant difference compared with the CRT3LFP group. The data are presented as the mean ± SEM. Statistical significance was determined by an unpaired two-tailed *t*-test, *n*=3 technical replicates.

Table S1. Antibodies used in this study

| **Ab ID** | **Ab description** | **Manufacturer/Catalogue number** | **Application** |
| --- | --- | --- | --- |
| Calreticulin (D2E6) XP Rabbit mAb | Monoclonal Ab | Cell Signaling/12238 | Elisa |
| Anti-Calreticulin | Monoclonal Ab | Thermal Fisher/MA5-15382 |  |
| L-asparaginase | Polyclonal antibody | Abcam/ab55824 |  |
| Calreticulin (D3E6) XP® Rabbit mAb (PE Conjugate) | Clone D3E6 | Cell Signaling/19780S | Flow cytometry |
| Rabbit (DA1E) mAb IgG XP® Isotype Control (PE Conjugate) | Clone DA1E | Cell Signaling/5742S |  |
| Anti-His tag | Monoclonal Ab | Abcam/ab245114 |  |
| Goat anti-rabbit secondary antibody,  Alexa Flour 488 |  | Thermo Scientific/A11008 |  |
| Anti-mouse CD16/32 | TruStain FcX™ Fc blocking solution | BioLegend/101320 |  |
| Live/dead Fixable aqua dead cell stain kit |  | Invitrogen/L34957 |  |
| Pacific Blue anti-mouse CD45.2 antibody | Specific monoclonal Ab | BioLegend/109820 |  |
| Anti-mouse CD3-FITC | Clone 17A2 | eBioscience/11-0032-82 |  |
| Anti-mouse 11b-FITC | Clone M1/70 | BioLegend/101205 |  |
| Anti-mouse CD8a-PE | Clone 53-6.7 | eBioscience/12-0081-82 |  |
| PE anti-mouse CD172a (SIRPα) Antibody (PE Conjugate) | Clone P84 | Biolegend/144012 |  |
| Rat IgG1, κ Isotype Ctrl Antibody (PE Conjugate) | Clone RTK2071 | Biolegend/400408 |  |
| Anti-mouse CD47 Antibody (PE Conjugate) | Clone miap301 | Biolegend/127508 |  |
| Rat IgG2a, κ Isotype Ctrl Antibody (PE Conjugate) | Clone RTK2758 | Biolegend/ 400508 |  |
| Anti-mouse CD4-APC | Clone GK1.5 | BioLegend/100412 |  |
| Anti-mouse CD49-APC | Clone DX5 | BioLegend/108909 |  |
| Anti-mouse CD206-APC | Clone B7-H1 | BioLegend/C068C2 |  |
| Anti-mouse CD11c-APC | Clone N418 | Biolegend/117310 |  |
| Anti-mouse granzyme B-PerCP-Cy5.5 | Clone QA16A02 | BioLegend/372212 |  |
| Anti-mouse CD62L-PerCP-Cy5.5 | Clone MEL-14 | BioLegend/104432 |  |
| Anti-mouse MHCII-PerCP-Cy5.5 | Clone M5/114.15.2 | BioLegend/107625 |  |
| Anti-mouse Ly-6G-PerCP-Cy5.5 | Clone RB6-8C5 | BioLegend/127615 |  |
| Anti-mouse F4/80-PerCP-Cy5.5 | Clone BM8 | BioLegend/123128 |  |
| Anti-mouse Ki67-PE-Cy7 | Clone SolA15 | Thermo Fisher Scientific/25469882 |  |
| PE/Cy7 anti-mouse CD152 | Clone UC10-4B9 | BioLegend/106313 |  |
| Anti-mouse CD8 PE-Cy7 | Clone 53-6.7 | eBioscience/25-0081-82 |  |
| Anti-mouse CD11c PE-Cy7 | Clone N418 | Thermo Fisher Scientific/25-0114-82 |  |
| Anti-mouse PD-1-APC-Cy7 | Clone 29F.1A12 | BioLegend/135224 |  |
| Anti-mouse CD44-APC-Cy7 | Clone IM7 | BioLegend/103028 |  |
| Anti-mouse CD86-APC-Cy7 | Clone GL-1 | BioLegend/105029 |  |
| Anti-mouse CD8α | Clone 2.43 | Bio X Cell/BP0061 | CD8^+^ T depletion |
| Anti-mouse CD47 | Clone MIAP301 | Bio X Cell/BE0270 | In vivo CD47 blockade |
| Recombinant anti-6X His tag antibody |  | Abcam/ab245114 | Western blotting |
| L-asparaginase | Polyclonal antibody | Abcam/ab55824 |  |
| Rabbit anti-FlaB | Polyclonal Ab. | A gift from Dr. Joon Haeng Rhee, Chonnam National University Medical school. |  |
| Anti-rabbit secondary antibody, HRP |  | Invitrogen/31460 |  |
| Asparaginase antibody (HRP) |  | GenTex/GTX40848 |  |
| Goat anti-rabbit secondary antibody Alexa Flour 488 |  | Thermo scientific/A110088 | Confocal immunofluorescence imaging |
| Wheat germ agglutinin-Alexa Flour 555 Conjugate |  | Thermo scientific/W32464 |  |

Table S2. CDI value of CRT3LFP combined with CNC018 bacteria in CT26 and MC38 tumor models

| **Average tumor volume (mm^3^)** | **Control**  **(PBS)** | **A**  **(CNC018)** | **B**  **(CRT3LFP)** | **AB**  **(CNC018 + CRT3LFP)** | **CDI** | **Interpretation** |
| --- | --- | --- | --- | --- | --- | --- |
| CT26 tumor volume (day 15) | 1661.725 | 1063.05 | 1241.525 | 377.45 | 0.475 | Synergism |
| MC38 tumor volume (day 9) | 983.67 | 337.75 | 357.25 | 108.5 | 0.885 | Synergism |

CDI, coefficient of drug interaction; CDI = (AB/control)/[(A/control) x (B/control)]
